# Interaction of Glutathione with MMACHC Arginine-Rich Pocket Variants Associated with Cobalamin C Disease: Insights from Molecular Modeling

**DOI:** 10.3390/biomedicines11123217

**Published:** 2023-12-04

**Authors:** Priya Antony, Bincy Baby, Amanat Ali, Ranjit Vijayan, Fatma Al Jasmi

**Affiliations:** 1 Department of Biology, College of Science, United Arab Emirates University, Al Ain P.O. Box 15551, United Arab Emirates; 2 Department of Genetics and Genomics, College of Medicine and Health Sciences, United Arab Emirates University, Al Ain P.O. Box 15551, United Arab Emirates; 3 The Big Data Analytics Center, United Arab Emirates University, Al Ain P.O. Box 15551, United Arab Emirates; 4 Zayed Center for Health Sciences, United Arab Emirates University, Al Ain P.O. Box 15551, United Arab Emirates; 5 Department of Pediatrics, Tawam Hospital, Al Ain P.O. Box 15258, United Arab Emirates

**Keywords:** glutathione, methylcobalamin, arginine-rich pocket, docking, molecular dynamics

## Abstract

Methylmalonic aciduria and homocystinuria type C protein (MMACHC) is required by the body to metabolize cobalamin (Cbl). Due to its complex structure and cofactor forms, Cbl passes through an extensive series of absorptive and processing steps before being delivered to mitochondrial methyl malonyl-CoA mutase and cytosolic methionine synthase. Depending on the cofactor attached, MMACHC performs either flavin-dependent reductive decyanation or glutathione (GSH)-dependent dealkylation. The alkyl groups of Cbl have to be removed in the presence of GSH to produce intermediates that can later be converted into active cofactor forms. Pathogenic mutations in the GSH binding site, such as R161Q, R161G, R206P, R206W, and R206Q, have been reported to cause Cbl diseases. The impact of these variations on MMACHC’s structure and how it affects GSH and Cbl binding at the molecular level is poorly understood. To better understand the molecular basis of this interaction, mutant structures involving the MMACHC-MeCbl-GSH complex were generated using in silico site-directed point mutations and explored using molecular dynamics (MD) simulations. The results revealed that mutations in the key arginine residues disrupt GSH binding by breaking the interactions and reducing the free energy of binding of GSH. Specifically, variations at position 206 appeared to produce weaker GSH binding. The lowered binding affinity for GSH in the variant structures could impact metabolic pathways involving Cbl and its trafficking.

## 1. Introduction

Vitamin B12 (cobalamin, Cbl) is a water-soluble micronutrient that is vital for human health [1]. Cbl must be obtained from the diet or supplements as the human body is unable to synthesize it. The three major chemical forms of Cbl obtained from the diet are hydroxocobalamin (HOCbl), methylcobalamin (MeCbl), and adenosylcobalamin (AdoCbl), while cyanocobalamin (CNCbl) is frequently taken as a supplement [2]. However, these chemical forms must first be processed by methylmalonic aciduria and homocystinuria type C (MMACHC) protein, also referred to as CblC. Cbl undergoes an elaborate sequence of absorptive and processing stages due to its complex structure and cofactor forms before it is delivered to mitochondrial methyl malonyl-CoA mutase (MUT, EC 5.4.99.2) or cytosolic methionine synthase (MS, EC 2.1.1.13). 

**Figure 1:**
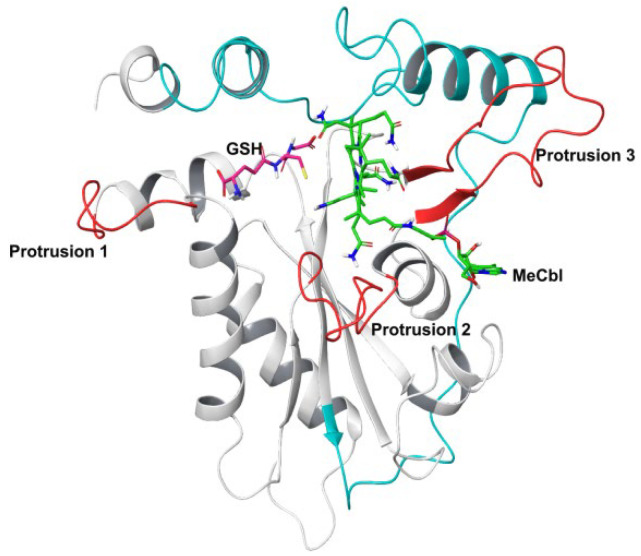
Structure of MMACHC-GSH-MeCbl complex. N-terminal is shown in white and C-terminal in cyan in colored cartoon representation. Protrusions 1, 2, and 3 are shown in red. Bound GSH is shown in pink and MeCbl in green in stick representation.

The primary sequence of MMACHC is well conserved among mammals and it contains 282 amino acids (Figure 1). The N terminal region (residues 1–172) and C terminal region (residues 183–244) are connected by a linker (residues 173–182). The N terminal core region consists of a four-stranded antiparallel β-sheet flanked by α-helices and a short antiparallel two-stranded β-sheet. The C-terminal module is made up of four α-helices that are attached to the core N-terminal module [3]. It has a vitamin B12-binding motif made up of the residues 122-HXXGX-126-154-GG-156 [4].

Any defects in the trafficking proteins will result in Cbl deficiency, which eventually leads to cobalamin disorders. These are inherited as autosomal recessive disorders and are divided into nine genetic complementation groups (*cblA*-*cblX*) [5]. Out of these groups, the most prevalent is CblC disease, caused by mutations in the *MMACHC* gene affecting the synthesis of AdoCbl and MeCbl, which results in combined methylmalonic aciduria (MMA) and homocystinuria (HCU) [6,7]. As MeCbl and AdoCbl are two essential coenzymes of MS and MUT enzymes, the absence of these causes an increase in methylmalonic acid and homocysteine in body fluids, as well as a decrease in methionine. As mentioned, CblC disease is the most frequent form found among patients with two distinctive phenotypic clinical forms with differing severity and age of onset: early-onset (EO) and late-onset disease (LO) [8]. In the EO form, the appearance of symptoms such as progressive encephalopathy, seizures, hypotonia, and megaloblastic anemia are observed within the first year of life, which aggravates the severity of the disease and causes failure to thrive. Patients with the LO form possess relatively milder clinical symptoms with acute neurological symptoms, neuropsychiatric symptoms, and thromboembolic complication [9,10,11].

Previous studies have implicated the role of *MMACHC* gene mutations in the development of CblC deficiency [12]. This gene encodes the 282 amino acid MMACHC protein, which is essential for Cbl metabolism. The sequence of this protein is well-conserved across mammals and it contains an unusual vitamin B12-binding motif [6]. MMACHC is able to bind a variety of Cbl forms with different upper axial ligands, including AdoCbl, MeCbl, CNCbl, and OHCbl (Supplementary Figure S1) [3]. Depending on the cofactor bound, MMACHC performs a dual function; it can either perform flavin mononucleotide (FMN)-dependent reductive decyanation of CNCbl or glutathione (GSH)-dependent dealkylation of alkyl cobalamins [13,14]. Reductive decyanation products include cyanide and cob(II)alamin. Alkyl cobalamins (RCbl) are cleaved via a nucleophilic displacement reaction in the presence of GSH, producing glutathione thioether and cob(I)alamin, which is rapidly oxidized to either cob(II)alamin or aquocobalamin (OH2Cbl) under aerobic conditions [15]. Therefore, the removal of alkyl groups by GSH or FMN is a prerequisite for generating intermediates that can be subsequently converted into active cofactor forms. MMACHC occurs largely as a monomer in the apo state. When unprocessed Cbl along with the upper axial ligand (GSH) binds to MMACHC it could switch to a homodimeric state. Following the removal of the upper axial ligand by GSH, MMACHC is available for interaction with MMADHC (methylmalonic aciduria and homocystinuria type D protein), forming a trafficking chaperone delivering cobalamin to client enzymes [16]. This interaction is crucial for cobalamin processing, and mutations in either protein can interfere with complex formation, leading to cobalamin related diseases.

Several pathogenic variants of the *MMACHC* gene have been reported to cause combined methylmalonic acidemia with homocystinuria [17]. The most commonly identified mutations include c.271dupA, c.394C > T, and c.331C > T. The mutations c.271dupA and c.331C > T have been associated with EO disease, and c.394C > T is primarily associated with LO disease [6,18,19]. Apart from this, a wide range of missense mutations in the *MMACHC* gene have been identified that have an impact on protein stability as well as Cbl and GSH binding [20]. The vast majority of reported missense mutations are concentrated in the Cbl binding region and are likely to interfere with Cbl binding. However, mutations outside the Cbl binding site have also been reported to cause CblC disorders. For instance, the R161Q missense mutation has been linked to CblC disease in various ethnic groups [21]. This highly conserved residue is located in a cavity close to the Cbl binding region where GSH binds [22]. The entire Cbl trafficking pathway may be affected by mutations in these areas since the binding of GSH is necessary for removing the alkyl groups of Cbl. Along with the R161 residue, this cavity is lined by Arg206 and Arg230 residues, making it a pocket dense in positively charged residues [22]. Although R161Q, R161G, R206Q, R206P, and R206W mutations in this cavity are associated with both mild and severe pathogenic diseases, sufficient structural data do not exist to assess how these mutations affect GSH and Cbl binding at the molecular level.

*MMACHC* mutations are associated with a spectrum of clinical manifestations, emphasizing the significance of this gene in human health. Thus, the main objective of this study was to study the structural impact of mutations in the GSH binding site. Such studies could provide a deeper understanding of the pathogenic mechanisms, as well as potentially help in developing targeted therapeutic interventions for this disorder. A deeper understanding of wild-type (WT) and mutant MMACHC with GSH and Cbl could shed light on its structure-function relationships and disease severity. Molecular dynamics (MD) simulations are an invaluable tool for investigating mutation-induced variations in protein structure, function, and interaction at the atomic level. As mutant protein structures of MMACHC are currently not available, these were modeled in this study using the three-dimensional structure of WT MMACHC. Subsequently, MD simulations of MMACHC-MeCbl-GSH complexes were performed to study the structural stability, interactions, and energetics of the binding of ligands. Using the MD trajectories of the systems, the binding free energy of the complexes was calculated using the molecular mechanics–generalized Born surface area (MM-GBSA) approach, and the role of key residues that stabilize GSH binding was established. In the absence of clinical or structural data, the results from this study provide an in-depth understanding of the structural stability and disease mechanism of this multifunctional enzyme.

## 2. Materials and Methods

### 2.1. Protein Structure Preparation and Mutant Modeling

The crystal structures of the MMACHC protein with MeCbl (PDB ID: 3SC0) and MMACHC with GSH (PDB ID: 5UOS) were retrieved from the Protein Data Bank (PDB) [3,23]. The MMACHC-MeCbl-GSH complex was modeled by performing flexible ligand docking of MeCbl with 5UOS using Schrödinger Glide (Schrödinger release 2021-1, Schrödinger, LLC, New York, NY, USA). For this procedure, a receptor grid was generated on the prepared protein (5UOS). An OPLS-AA 2005 force field was used to model the protein and ligand. The van der Waals radii of the protein atoms were scaled by 1.0, and the charge cutoff for polarity was 0.25. The grid box was set at the center of the Cbl binding region to accommodate the MeCbl ligand. Molecular docking was performed using Schrödinger Glide employing the extra precision (XP) mode. Docked poses were scored and ranked using the GlideScore scoring function [24]. Five mutants (R161G, R161Q, R206P, R206W, R206Q) were generated by employing computational point mutations using the residue mutation panel of Schrodinger Maestro. The Schrödinger Protein Preparation Wizard was used to prepare the WT and mutant structures. This included assigning suitable bond ordering, adding disulfide bonds, modifying ionization states, fixing disorientated groups, capping termini, adding sidechains and missing atoms, assigning partial charges, and eliminating undesirable metals, cofactors, and water molecules. Hydrogen atoms were also added, and a conventional protonation condition at pH 7 was employed. Finally, hydrogen atoms were optimized, and restrained minimization was employed to obtain stable structures [25].

### 2.2. Molecular Dynamics Simulations 

The WT and mutant MMACHC-MeCbl-GSH complex were subjected to MD simulations to assess the strength and stability of the intermolecular interactions. Six simulation systems (WT, R161G, R161Q, R206P, R206W, and R206Q) were established, and simulations of 250 ns were performed in triplicate. All complexes were placed in an orthorhombic box of size 69 Å × 69 Å × 69 Å and solvated with single-point charge water molecules using the Desmond System Builder [26]. Subsequently, each system set-up was neutralized with counterions, and a salt concentration of 0.15 M NaCl was maintained. Desmond was used to run 250 ns MD simulations of the complexes [27]. Initial velocities, drawn from a Maxwell–Boltzmann distribution at 300 K, were assigned to each atom based on different random seeds. Prior to MD simulations, all systems underwent the steepest descent minimization and Desmond’s default eight-stage relaxation protocol. An NPT ensemble with the temperature at 300 K and the pressure at 1 atm was applied in all runs [28,29]. A Nosé–Hoover thermostat, with a relaxation time of 1 ps, and an isotropic Martyna–Tobias–Klein barostat, with a coupling constant of 2 ps, were used for this. Long-range Coulombic interactions were evaluated using the smooth particle mesh Ewald approach, and the short-range van der Waals and Coulombic interactions were calculated at a cut-off radius of 9.0 Å [30]. A time-reversible reference system propagator algorithm (RESPA) integrator was used with an inner time step of 2.0 fs and an outer time step of 6.0 fs [31]. All bonds involving hydrogen atoms were constrained using the M-SHAKE algorithm implemented in Desmond [32]. The OPLS-AA 2005 force field parameters were used in all simulations. Table S1 shows the atom types and parameters used for GSH and MeCbl. The binding free energy of the wild-type and the mutant complexes were computed based on the molecular mechanics–generalized Born surface area (MM-GBSA) approach by extracting frames at 5 ns intervals from MD simulation trajectories. The MM-GBSA-based binding free energy (ΔG_bind_) was then calculated using Schrödinger Prime and the VSGB 2.0 solvation model [33]. Packaged and custom scripts were used to analyze the simulation data.

## 3. Results and Discussions

### 3.1. MD Simulations of Wild-Type MMACHC with MeCbl and GSH

**Figure 2:**
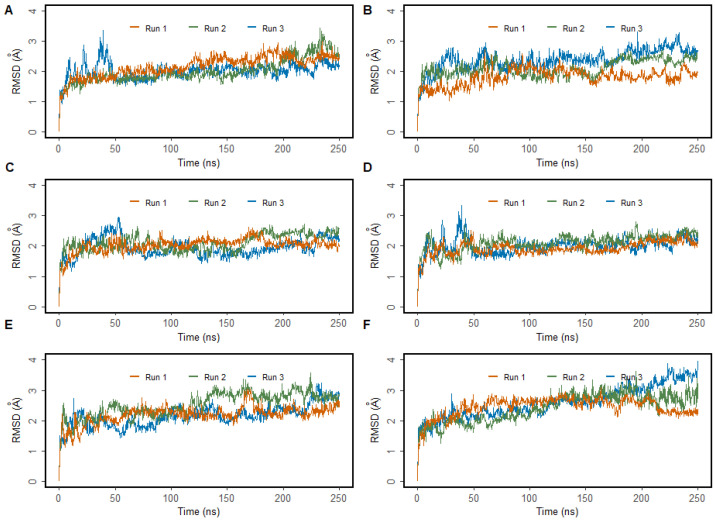
Root mean square deviation (RMSD) of MMACHC in MMACHC-MeCbl-GSH complexes obtained from 250 ns MD simulations. (**A**) WT MMACHC, (**B**) R161G mutant, (**C**) R161Q mutant, (**D**) R206P mutant, (**E**) R206W mutant, (**F**) R206Q mutant.

**Figure 3:**
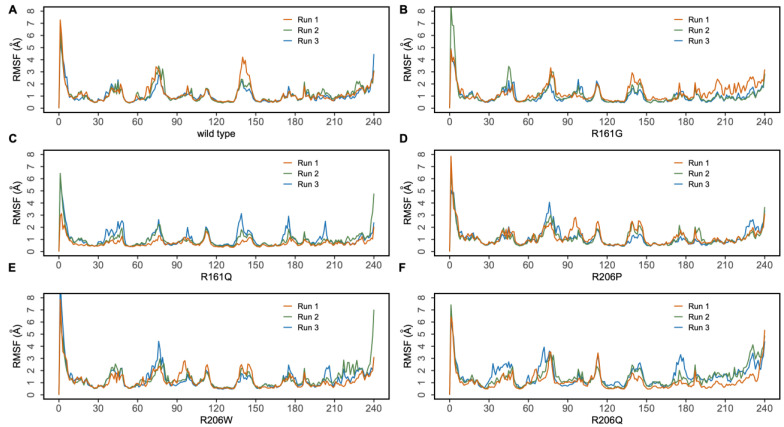
Root mean square fluctuations (RMSF) in MMACHC Cα atoms in the MMACHC-MeCbl-GSH complexes obtained from 250 ns MD simulations. (**A**) WT MMACHC, (**B**) R161G structure, (**C**) R161Q structure, (**D**) R206P structure, (**E**) R206W structure, (**F**) R206Q structure.

**Figure 4:**
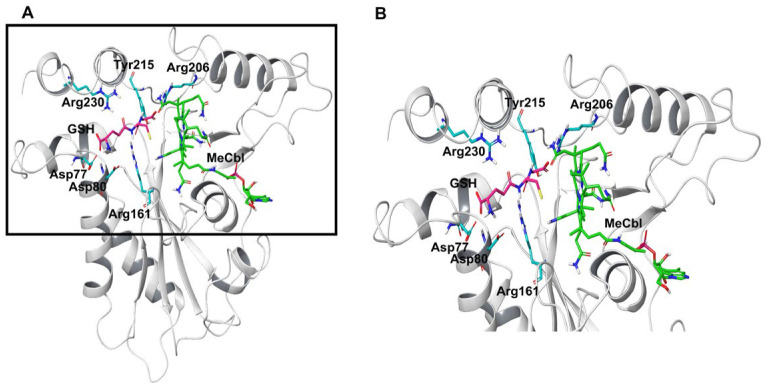
(**A**) WT MMACHC with GSH bound in the arginine-rich pocket. (**B**) Enlarged view of arginine-rich pocket shown in black box in (**A**).

**Figure 5:**
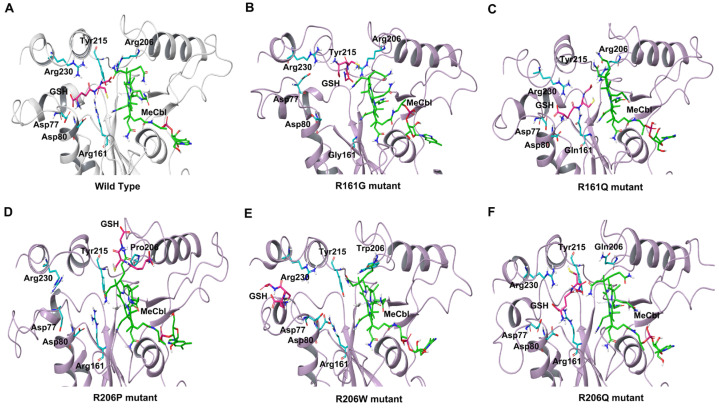
Interactions of GSH with residues in the arginine-rich pocket observed in the final frame of MD simulations. (**A**) WT, (**B**) R161G structure, (**C**) R161Q structure, (**D**) R206P structure, (**E**) R206W structure, (**F**) R206Q structure.

**Figure 6:**
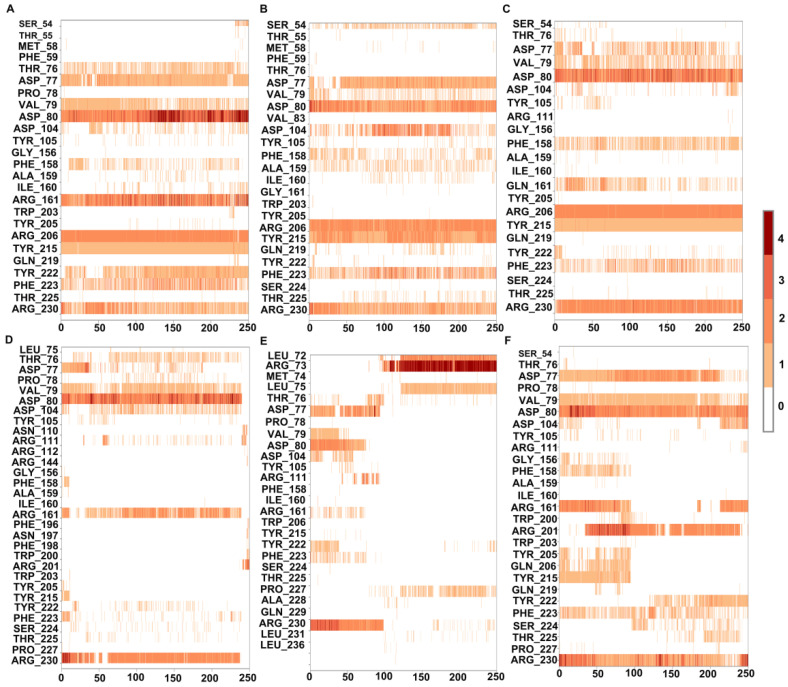
Interaction of GSH with MMACHC residues in the wild-type and mutated structures observed in 250 ns MD simulations. (**A**) Wild type, (**B**) R161G, (**C**) R161Q, (**D**) R206P, (**E**) R206W, (**F**) R206Q. The interacting residue is indicated on the y-axis. The number of contacts made is indicated by the scale on the right of the plot.

To understand the dynamics and stability of the WT MMACHC-MeCbl-GSH complex, 250 ns MD simulations were carried out in triplicate. Initially, the stability of the structure was evaluated by evaluating the movement of the protein backbone variation using root mean square deviation (RMSD). The RMSD rose slightly during the initial stage of the simulations, but stabilized below 3 Å within a few nanoseconds and stayed below this, indicating structural integrity in all three runs (Figure 2). The average RMSD value of three runs was observed to be 2.6 Å. In run 3, higher fluctuations were observed between 0 and 50 ns as the system attempted to reach equilibrium. Apart from this, higher fluctuations were caused due to the conformational fluctuations observed in the protrusion 1 (residues 69–77) region, which is involved in dimer formation. To investigate the residue level protein flexibility of each system, the root mean square fluctuation (RMSF) of protein Cα atoms was evaluated, which gives an indication of the mobility of each residue around its mean location. The RMSF plots demonstrated higher flexibility around the loop regions, especially where three protrusions (Pr) were observed (Pr1:69–77; Pr2:104–116; Pr3:128–150) (Figure 3 and Supplementary Figures S13 and S14). The longest protrusion along with the C-terminal domain of the protein form the Cbl binding pocket [22]. The radius of gyration (Rg) shows the structural compactness and stability of the molecules [34]. The Rg values of wild-type and mutant systems were computed from the generated MD trajectories of 250 ns (Supplementary Figure S15). The MMACHC-GSH complexes were also studied for their interaction profiles. In the MMACHC-GSH complex, an extensive network of interactions, including hydrogen bonds, hydrophobic contacts, ionic interactions, and water bridges, assisted in the binding of GSH in its binding pocket within MMACHC. GSH is a tripeptide composed of glutamate, cysteine, and glycine amino acids [35]. Based on earlier structural and biochemical investigations, the most important residues required for GSH binding include Arg161, Arg206, and Arg230 [3]. An extended network of protein–GSH interactions was observed in the GSH binding pocket holding GSH in place (Figure 4 and Figure 5A). Multiple hydrogen bonds, hydrophobic contacts, ionic bonds, and water bridges were found to interact with all three peptide moieties (Glu, Cys, Gly) of GSH and protein residues such as Asp77, Val79, Asp80, Arg161, Arg206, Tyr215, and Arg230. These interactions were observed to continue throughout the simulation duration (Figure 6A and Supplementary Figure S2). Moreover, Arg161 acts as a GSH anchor through interactions of its guanidinium group and carboxamide moiety of cysteine. Additionally, the other interactions identified strengthened GSH binding even further [23]. The free energy of binding (ΔG_bind_) of these ligands was calculated using the molecular mechanics–generalized Born surface area (MM-GBSA) approach. It was observed that MMACHC bound MeCbl in the three simulations with ΔG_bind_ values of –140.03 ± 14.92 kcal/mol, –146.06 ± 18.62 kcal/mol, and –140.95 ± 15.04 kcal/mol, while GSH bound with ΔG_bind_ values of –36.31 ± 21.43 kcal/mol, –34.29 ± 18.12 kcal/mol, and –33.38 ± 19.65 kcal/mol. 

### 3.2. Structural Insights into Pathogenic Mutations in MMACHC with MeCbl and GSH

#### 3.2.1. R161G and R161Q

Arginine 161 is a highly conserved amino acid residue found within a cavity near the Cbl site and has no association with cobalamin [36]. Adenosylcobalamin and methylcobalamin undergo a dealkylation process in which glutathione acts as an electron donor. It has been suggested that this arginine residue is crucial for the binding of glutathione, which, in turn, aids in the Cbl trafficking process [3,22]. Arg161 mutations are among the most prominent missense variants that result in CblC dysfunction, and depending on the type of substitution, these mutations are linked with either early (R161G) or late (R161Q) disease onset [36]. MD simulation, in triplicate, was employed to investigate the disruptive effects of R161G and R161Q mutations on the conformational stability of MMACHC and GSH binding. RMSD values for the Cα backbone of mutants were computed from the 250 ns simulations. The RMSD for the R161G and R161Q mutants is shown in Figure 2A,B. The WT structure had an RMSD of 2.6 Å. However, the R161G and R161Q structures exhibited higher RMSD values of 3.0 Å and 2.73 Å, respectively. The higher RMSD values in the two systems could be attributed to structural disturbances and conformational rearrangements. The variation in the RMSD values is attributed to the fluctuations observed in the Pr1 region and the subsequent α-helix (Figure 2B,C). Figure 3B,C depict the RMSF of the mutant systems. Similar to the WT, higher flexibility was observed around the loop regions, especially the three protrusions. Based on the literature, it was reported that these flexible loops may serve as a molecular foundation for reconfiguring the binding pocket and accommodating the binding of diverse Cbl ligands [22]. Relatively higher RMSF was observed in the Pr1:69–77 region, indicating higher flexibility in this region. The RMSF data indicate that the substitution of Arg161 with glycine or glutamine increased protein flexibility in this region. This flexibility is likely to reduce the stabilizing interactions of nearby residues. The structural compactness of protein in both mutant systems was evaluated in terms of the radius of gyration (Rg) (Supplementary Figure S15). Notable deviations in Rg were observed in the R161G mutant structure. Interactions that persisted between GSH and mutant protein structures for at least 30% of the simulation time were examined (Supplementary Figures S3 and S4). As expected, when Arg161 was mutated to R161G and R161Q, the native hydrogen bond that binds the GSH in the active region of the protein was disrupted (Figure 5B,C). This is evidenced by higher ligand RMSF values when compared to the wild-type protein. This conclusion is consistent with the observations that mutations at position 161 (to G or Q) reduce CblC’s affinity for GSH [37]. The substitution of a positively charged arginine residue with a smaller glycine residue causes severe alterations in the organization of the binding site, as well as its physicochemical characteristics, affecting protein stability. Additionally, it was reported that the R161G mutation is prone to aggregation at the normal body temperature of 37 °C. These changes are likely to contribute to early disease development. However, the arginine-to-glutamine substitution may not significantly affect the protein stability as both residues are amphipathic, containing hydrophobic and polar areas, which may explain the late onset of the disease [21]. The three R161G mutants with MeCbl exhibited ΔG_bind_ values of –140.78 ± 16.10 kcal/mol, –138.14 ± 18.43 kcal/mol, –131.61 ± 15.51 kcal/mol, while GSH had ΔG_bind_ values of –35.78 ± 20.97 kcal/mol, –24.69 ± 17.05 kcal/mol, and –26.13 ± 18.40 kcal/mol. In the R161Q mutant, the ΔG_bind_ values for MeCbl were found to be –145.53 ± 14.02 kcal/mol, –148.82 ± 13.18 kcal/mol, and –141.20 ± 14.54 kcal/mol in the three runs, while GSH exhibited –36.09 ± 16.44 kcal/mol, –26.54 ± 17.12 kcal/mol, and –33.82 ± 15.77 kcal/mol. The reduced binding affinity of GSH compared to the WT clearly demonstrates the impact of the mutation on GSH binding.

#### 3.2.2. R206W, R206P, and R206Q

MD simulations were also run to examine the effect of R206W, R206P, and R206Q mutations on the conformational stability of MMACHC and GSH binding. In the R206P system, after the initial fluctuations, the RMSD value of the protein Cα atoms stayed below 3 Å, suggesting a stable system (Figure 2D). Based on the RMSF values, most of the fluctuations were observed in the loop region (69–77). This region fluctuated slightly higher than WT (Figure 3D). The majority of GSH interactions with the arginine-rich pocket residues were broken, as expected. In the case of R206P and R206W, GSH was observed to interact with the residues near the arginine-rich pocket (Figure 5D,E). Interactions with the key residues R161 and R206 were found to be either weak or disturbed in R206P, R206W, and R206Q throughout the simulations (Figure 6D–F). In two runs of R206P, GSH was found to interact with Asp104, Arg201, and Pro227 found in the vicinity of the arginine-rich pocket. Furthermore, in the third run, GSH broke away from the residues in its binding site, indicating the significance of the arginine residues in binding the molecule. Similar to R206P, in the R206W system, the interaction with Arg161, Arg206, and Arg230 was broken or intermittently broken during the 250 ns simulations. Additional interactions with Leu72, Arg73, and Leu75 through hydrogen bonds and water bridges were noted. Interactions with Asp77, Asp80, and Tyr215 were also lost after around 20 ns of the simulation time (Figure 6E). Interestingly, a weaker interaction with GSH was observed when the arginine at position 206 was mutated to proline or tryptophan when compared to R161G and R161Q (Figure 6). Interactions retained for over 30% of the simulation time were examined (Supplementary Figures S5–S7). In the R206Q system, protein RMSD fluctuations in the first two runs stayed below 3.5 Å, whereas in the final run, the protein deviated from its initial structure with conformational transitions. This was mainly due to the fluctuations observed in the end loops of the C terminal region. Analyzing ligand RMSD helps to identify ligand transitions occurring during the simulation. GSH was noted to exhibit higher fluctuations in mutant simulations as some of the protein–ligand contacts were lost (Supplementary Figure S8). Several intermolecular interactions were found to be broken or weak during the 250 ns simulations. In R206Q, similar to R206P, GSH detached from the binding site, strongly supporting weak binding in these mutants. Based on the RMSF and protein–ligand interaction analysis, larger fluctuations were observed in the loop (66–77) region of R206P, R206W, and R206Q mutants when compared to the WT and mutations at position 161 (Figure 3D–F). This correlates with the values of Rg generated for GSH in each complex. Even though R161 anchors the GSH in the arginine-rich pocket, R206 also plays a critical role in the binding of GSH. The protein Rg of all three mutant systems, particularly R206Q, deviated more when compared to the stable Rg observed in the WT structure (Supplementary Figure S15).

Free energy calculations were employed to further quantitatively compare the changes in interaction in the WT and mutated systems (Table 1). The WT system exhibited the most favorable binding free energy when compared to the mutants, suggesting the importance of the arginine residues in GSH binding. Arginine’s guanidino group’s high positive charge is shared via resonance with the three nitrogen atoms, making this group a very good binder of anionic groups. The replacement of this arginine with polar-neutral tryptophan reduced the ΔG_bind_ of GSH (–24.40 ± 20.36 kcal/mol, –28.53 ± 15.47 kcal/mol, and –15.61 ± 13.94 kcal/mol). Similar to this, a mutation to proline (R206P) reduces the ΔG_bind_ of GSH (–22.62 ± 14.31 kcal/mol, –25.66 ± 16.48 kcal/mol, and –20.68 ± 16.86 kcal/mol). It was observed that a mutation to glutamine drastically affected the ΔG_bind_ of GSH (–12.95 ± 10.10 kcal/mol, –11.90 ± 11.70 kcal/mol, –10.75 ± 8.87 kcal/mol). Based on these calculations, mutations at position 206 appear to have the greatest impact and weaker GSH binding than the other mutants. 

To evaluate the stability and binding characteristics, the structural and physical properties of GSH, including RMSD, radius of gyration (Rg), polar surface area (PSA), solvent-accessible surface area (SASA), and molecular surface area (MolSA), were also assessed in all simulation systems (Supplementary Figures S8–S12). Ligand RMSD is an indicator of ligand stability in a protein’s active site. The RMSD of GSH in the WT, R161G, and R161Q variants was found to be less than 2 Å, whereas it was higher in the R206Q, R206P, and R206W structures (Supplementary Figure S8). This suggests that variations at position 161 have a greater impact on GSH binding. Ligand Rg measures the compactness of a ligand; the Rg of GSH in the WT remained constant throughout the duration of the simulations, indicating that the ligand did not undergo any significant conformational change (Supplementary Figure S9A). Fluctuations were observed in the mutants, indicating conformational changes in GSH, and therefore its binding and stability (Supplementary Figure S9B–F). The intermolecular hydrogen bonds of GSH and the MMACHC complex throughout the simulation were analyzed. The polar, molecular, and solvent-accessible surface area of GSH exhibited notable fluctuations in the mutant structures when compared to the WT (Supplementary Figures S10–S12). Variations at position 206 exhibited high SASA values as GSH detached from the binding site during the simulations. It was observed that hydrogen bonds remained intact in the wild type, whereas in mutants, especially at position 206, most of the crucial interactions were found to be broken as they detach from the binding region (Supplementary Figure S16). From the trajectory, snapshots of GSH interacting with the protein are provided (Supplementary Figures S17–S22). Larger displacements and detachment of the ligand are visible in structures with variations at position 206. Based on the simulation data and binding free energy calculations, mutations at position 206 had a more severe impact on GSH binding. This would directly affect the dealkylation of Cbl, which may eventually cause CblC disease. 

## 4. Conclusions

To provide insight into the structural dynamics of MeCbl and GSH bound to MMACHC and the related disease mechanisms of mutants, MD simulations were performed on WT and mutated systems. In the WT system, protein–GSH contacts identified in the crystal structure were faithfully retained in multiple MD simulations. As the binding of GSH is essential for the dealkylation reaction of MMACHC, mutations in the arginine-rich pocket where GSH binds will impair MMACHC transport. In the absence of structural data related to the variants, the results presented here show that mutations in key arginine residues responsible for GSH binding disrupt GSH binding. Variations at both R161 and R206 have a negative effect on GSH binding. However, based on the simulations, variations at position 206 appear to produce weaker GSH binding than the other variants studied. These mutations could affect the utilization of GSH and the dealkylation process, contributing to its pathogenicity. In conclusion, in silico modeling and simulations indicate that GSH’s binding affinity to MMACHC is significantly reduced in mutants involving the arginine-rich binding cavity where it binds. Future structural studies could provide further insights to confirm these findings. Additionally, investigating the impact of MMACHC mutations and their correlation with clinical outcomes will provide valuable insights into the pathophysiology of CblC disease and guide the development of targeted therapies. 

## Figures and Tables

**Table 1:** Computed free energy of binding (ΔG_bind_) of Cbl and GSH in their binding sites in WT and mutant MMACHC structures.

Wild Type	MeCbl (kcal/mol)	GSH (kcal/mol)
Run 1	–140.03 ± 14.92	–36.31 ± 21.43
Run 2	–146.06 ± 18.62	–34.29 ± 18.12
Run 3	–140.95 ± 15.04	–33.38 ± 19.65
**R161G**	**MeCbl** **(kcal/mol)**	**GSH** **(kcal/mol)**
Run 1	–140.78 ± 16.10	–35.78 ± 20.97
Run 2	–138.14 ± 18.43	–24.69 ± 17.05
Run 3	–131.61 ± 15.51	–26.13 ± 18.40
**R161Q**	**MeCbl** **(kcal/mol)**	**GSH** **(kcal/mol)**
Run 1	–145.53 ± 14.02	–36.09 ± 16.44
Run 2	–148.82 ± 13.18	–26.54 ± 17.12
Run 3	–141.20 ± 14.54	–33.82 ± 15.77
**R206P**	**MeCbl** **(kcal/mol)**	**GSH** **(kcal/mol)**
Run 1	–144.50 ± 13.60	–22.62 ± 14.31
Run 2	–155.96 ± 17.16	–25.66 ± 16.48
Run 3	–145.21 ± 14.16	–20.68 ± 16.86

## Supplementary Materials

The following supporting information can be downloaded at: https://www.mdpi.com/article/10.3390/biomedicines11123217/s1, Figure S1: Chemical structure of the different forms of vitamin B12. Figure S2: Interactions between GSH and the wild-type structures retained for more than 30% of the simulation time. Figure S3: Interactions between GSH and the R161G structures retained for more than 30% of the simulation time. Figure S4: Interactions between GSH and the R161Q structures retained for more than 30% of the simulation time. Figure S5: Interactions between GSH and the R206P structures retained for more than 30% of the simulation time. Figure S6: Interactions between GSH and the R206W structures retained for more than 30% of the simulation time. Figure S7: Interactions between GSH and the R206Q structures retained for more than 30% of the simulation time. Figure S8: RMSD of GSH during 250 ns of the MD simulation. (A) WT, (B) R161G, (C) R161Q, (D) R206P, (E) R206W, and (F) R206Q. Figure S9: Radius of gyration (Rg) of GSH during 250 ns of the MD simulation. (A) WT, (B) R161G, (C) R161Q, (D) R206P, (E) R206W, and (F) R206Q. Figure S10: Molecular surface area (MolSA) of GSH during 250 ns of the MD simulation. (A) WT, (B) R161G, (C) R161Q, (D) R206P, (E) R206W, and (F) R206Q. Figure S11: Solvent-accessible surface area (SASA) of GSH during 250 ns of the MD simulation. (A) WT, (B) R161G, (C) R161Q, (D) R206P, (E) R206W, and (F) R206Q. Figure S12: Polar surface area (PSA) of GSH during 250 ns of the MD simulation. (A) WT, (B) R161G, (C) R161Q, (D) R206P, (E) R206W, and (F) R206Q. Figure S13: Protein structure rendered after RMSF from MD simulations projected as beta factors in MMACHC wild-type (WT) and R161G and R161Q mutants. Figure S14: Protein structure rendered after RMSF from MD simulations projected as beta factors in MMACHC R206P, R206W, and R206Q mutants. Figure S15: Radius of gyration (Rg) of MMACHC protein during 250 ns of the MD simulation. (A) WT, (B) R161G, (C) R161Q, (D) R206P, (E) R206W, and (F) R206Q. Figure S16: Number of protein–ligand hydrogen bonds observed during MD simulations. Figure S17: Snapshots of GSH taken at 0 ns (pink), 100 ns (cyan), 200 ns (yellow), and 250 ns (green) from the trajectory of GSH complexed with wild-type (WT) MMACHC. Figure S18: Snapshots of GSH taken at 0 ns (pink), 100 ns (cyan), 200 ns (yellow), and 250 ns (green) from the trajectory of GSH complexed with R161G. Figure S19: Snapshots of GSH taken at 0 ns (pink), 100 ns (cyan), 200 ns (yellow), and 250 ns (green) from the trajectory of GSH complexed with R161Q. Figure S20: Snapshots of GSH taken at 0 ns (pink), 100 ns (cyan), 200 ns (yellow), and 250 ns (green) from the trajectory of GSH complexed with R206P. Figure S21: Snapshots of GSH taken at 0 ns (pink), 100 ns (cyan), 200 ns (yellow), and 250 ns (green) from the trajectory of GSH complexed with R206W. Figure S22: Snapshots of GSH taken at 0 ns (pink), 100 ns (cyan), 200 ns (yellow), and 250 ns (green) from the trajectory of GSH complexed with R206Q. Table S1: Atom types and parameters used for GSH and MeCbl.
