# Supplementary material for: Interaction of Glutathione with MMACHC Arginine-Rich Pocket Variants Associated with Cobalamin C Disease: Insights from Molecular Modeling
Source: Biomedicines. 2023 Dec 4;11(12):3217. doi: 10.3390/biomedicines11123217 (PMC10740964; doi:10.3390/biomedicines11123217)
Supplement: Supplementary file 1 [file biomedicines-11-03217-s001.zip › biomedicines-2652421-supplementary.pdf]

Interaction of glutathione with MMACHC arginine-rich pocket variants associated with cobalamin C disease: Insights from molecular modeling

Priya Antony[1,†], Bincy Baby[1,†], Amanat Ali[2], Ranjit Vijayan[1,3,4,*], Fatma Al Jasmi[2,5,*]

[1] Department of Biology, College of Science, United Arab Emirates University, PO Box 15551, Al Ain, United Arab Emirates
[2] Department of Genetics and Genomics, College of Medicine and Health Sciences, United Arab Emirates University, PO Box 15551, Al Ain, United Arab Emirates
[3] The Big Data Analytics Center, United Arab Emirates University, PO Box 15551, Al Ain, United Arab Emirates.
[4] Zayed Center for Health Sciences, United Arab Emirates University, PO Box 15551, Al Ain, United Arab Emirates.
[5] Department of Pediatrics, Tawam Hospital, Al Ain, United Arab Emirates

† These authors contributed equally to this work.
* Corresponding authors: ranjit.v@uaeu.ac.ae (R.V.) and aljasmif@uaeu.ac.ae (F.A.J.)

Supplementary Materials

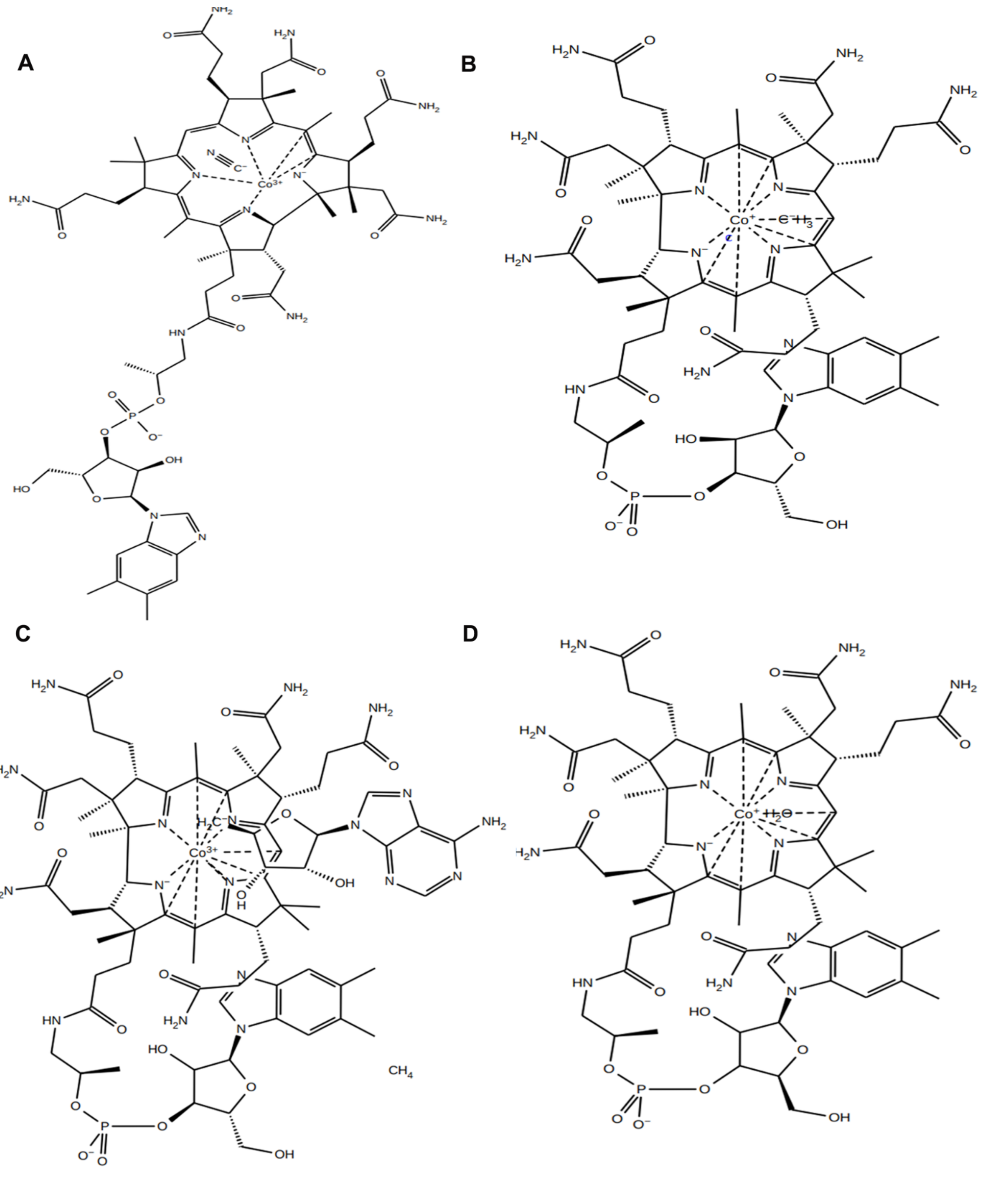

Figure S1: Chemical Structure of different forms of vitamin B12. A) Cyanocobalamin (CNCbl) B) Methylcobalamin (MeCbl) C) Adenosylcobalamin (AdoCbl) D) Hydroxocobalamin (OHCbl).

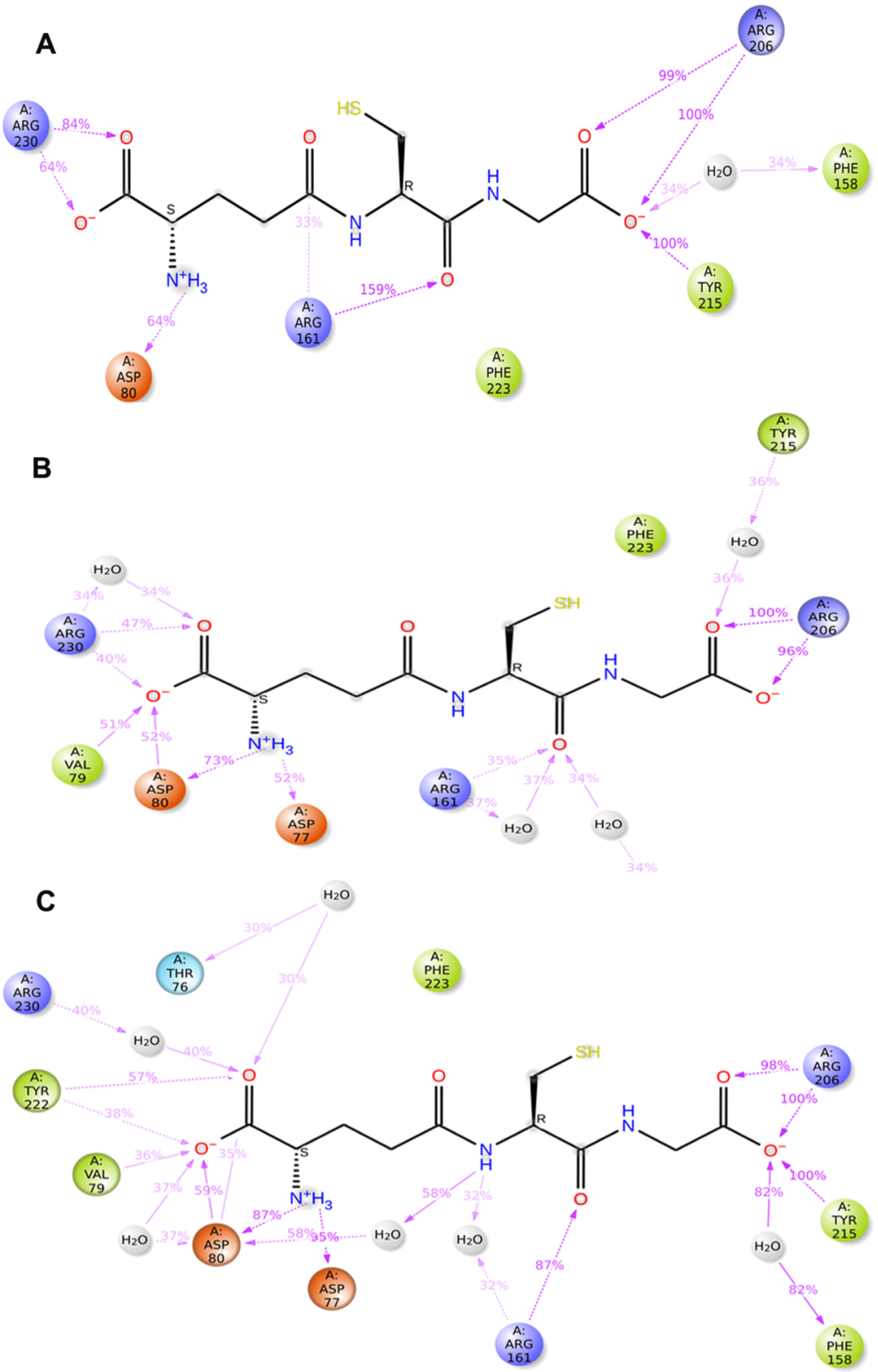

Figure S2: Interactions between GSH and the wild type structures retained for more than 30% of the simulation time. A) run 1 B) run 2 C) run 3.

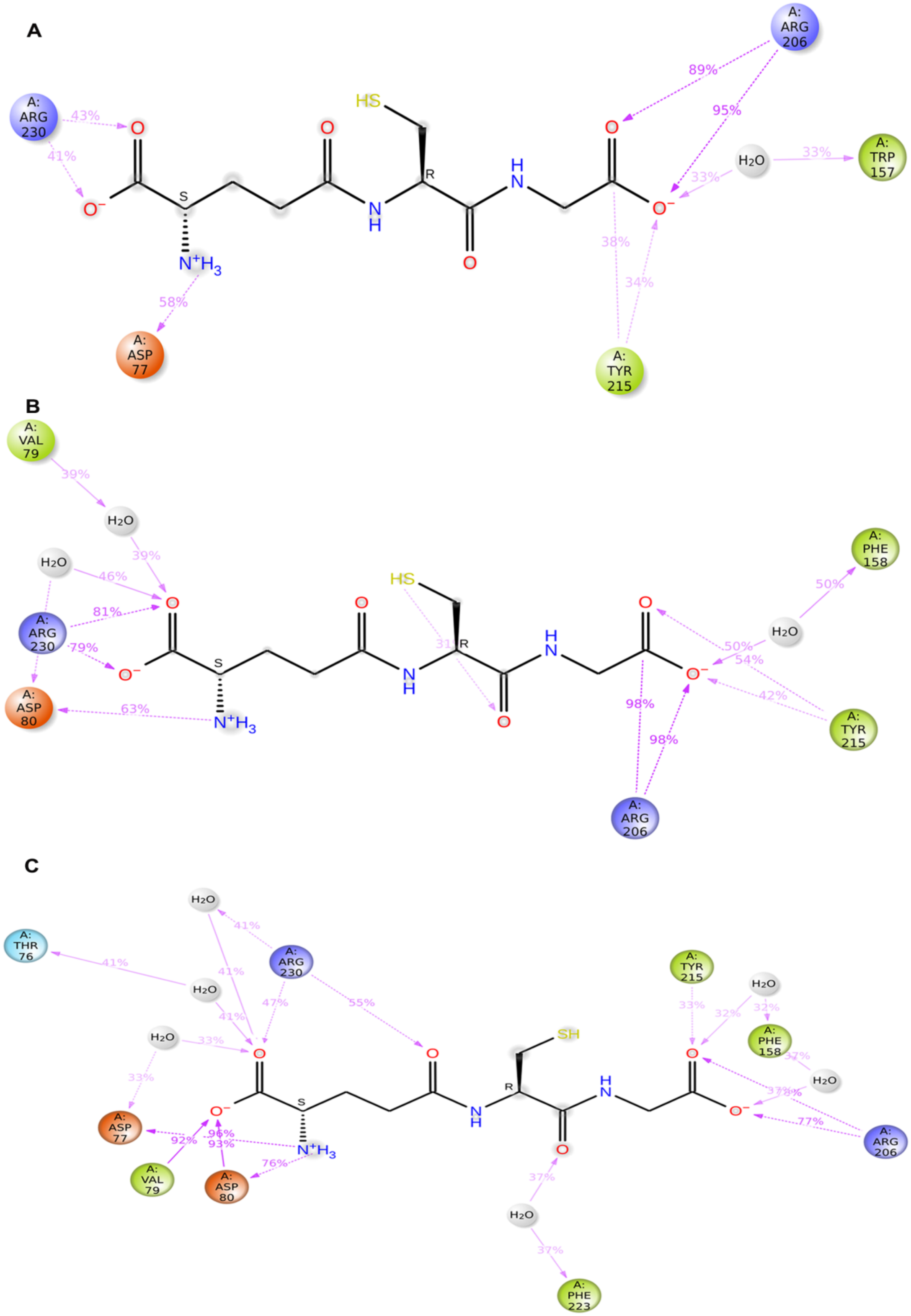

Figure S3: Interactions between GSH and the R161G structures retained for more than 30% of the simulation time. A) R161G run1 B) R161G run2 C) R161G run3.

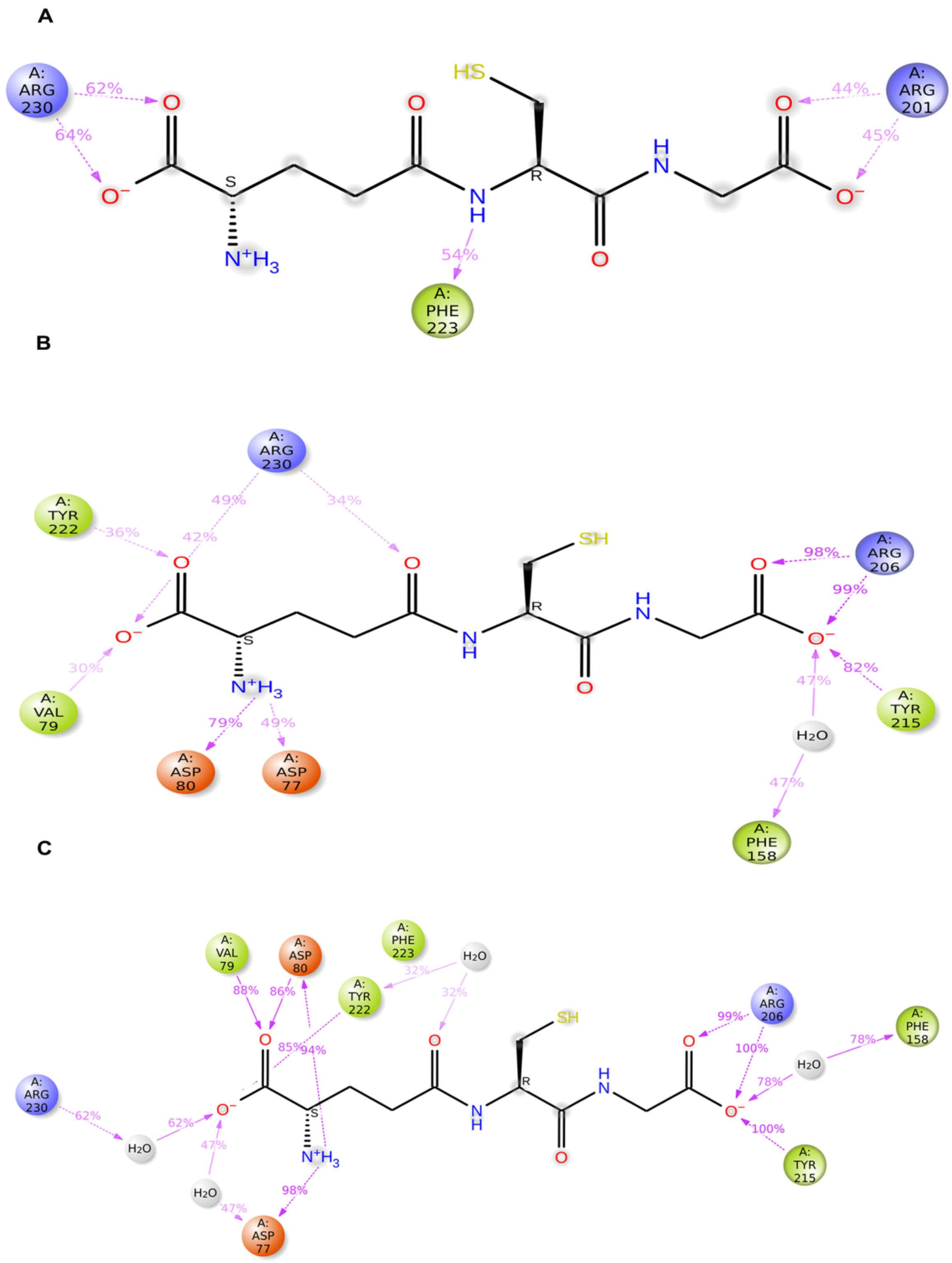

Figure S4: Interactions between GSH and the R161Q structures retained for more than 30% of the simulation time. A) R161Q run 1; B) R161Q run 2; C) R161Q run 3.

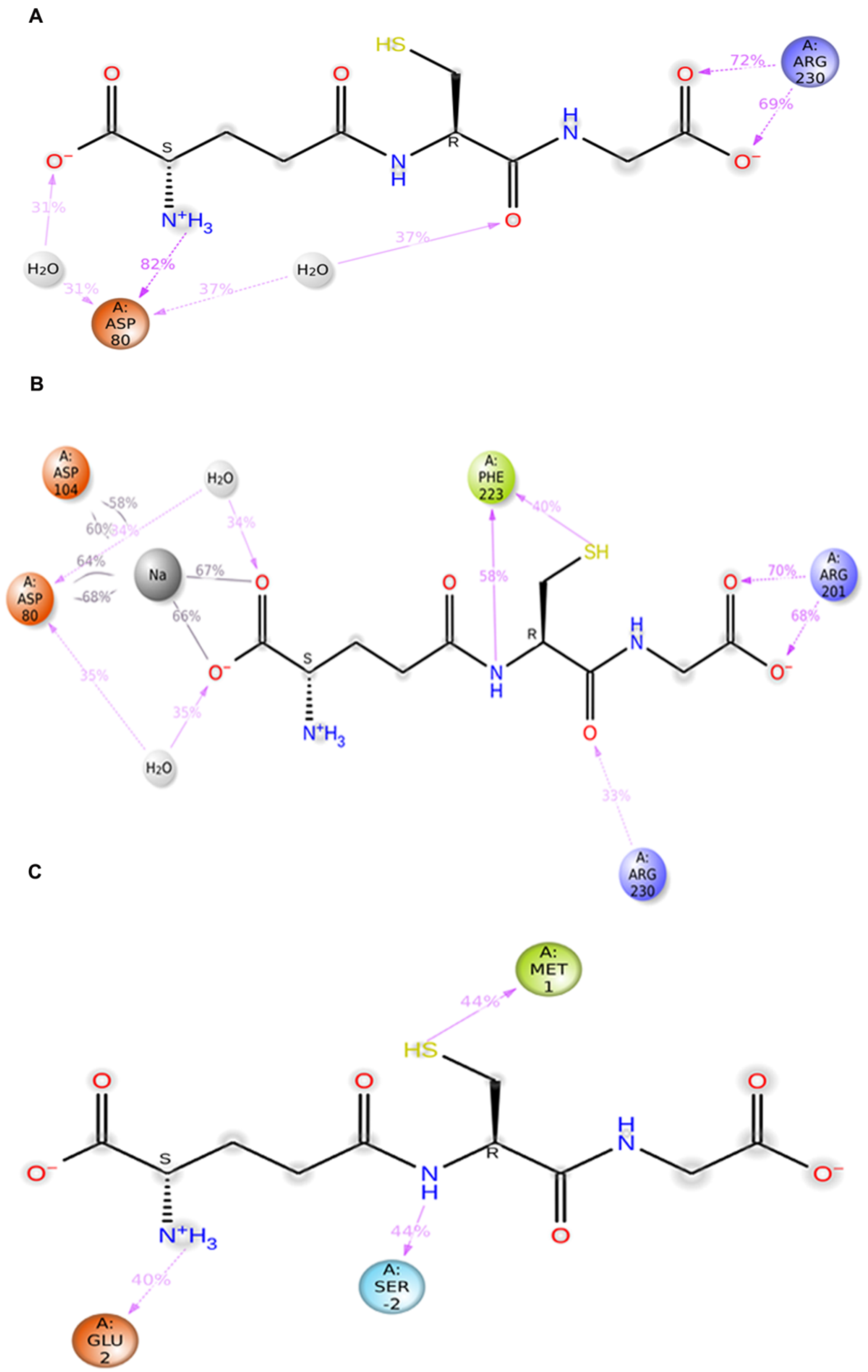

Figure S5: Interactions between GSH and the R206P structures retained for more than 30% of the simulation time. A) R206P run 1; B) R206P run 2; C) R206P run 3.

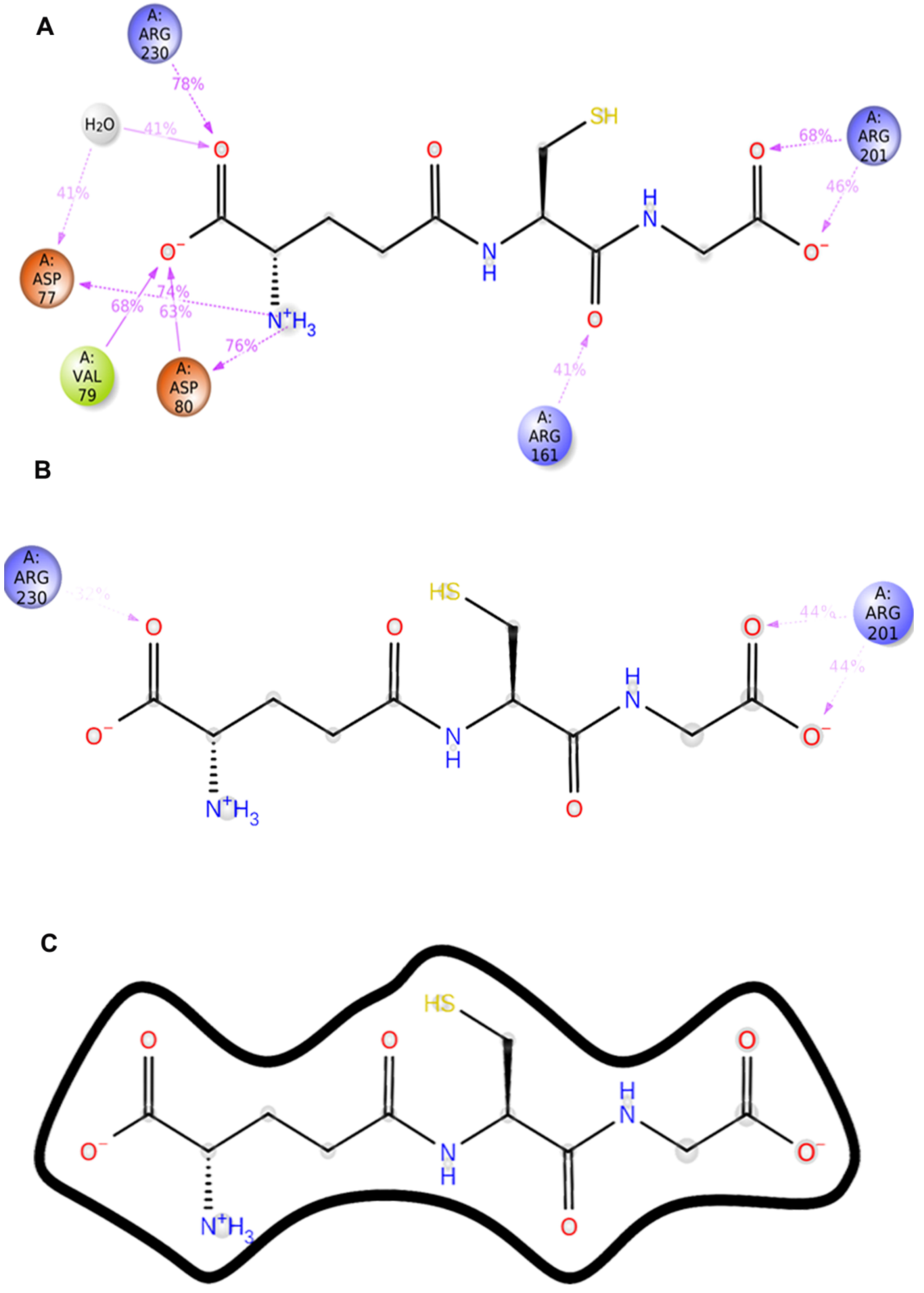

Figure S6: Interactions between GSH and the R206W structures retained for more than 30% of the simulation time. A) R206W run 1; B) R206W run 2; C) R206W run 3.

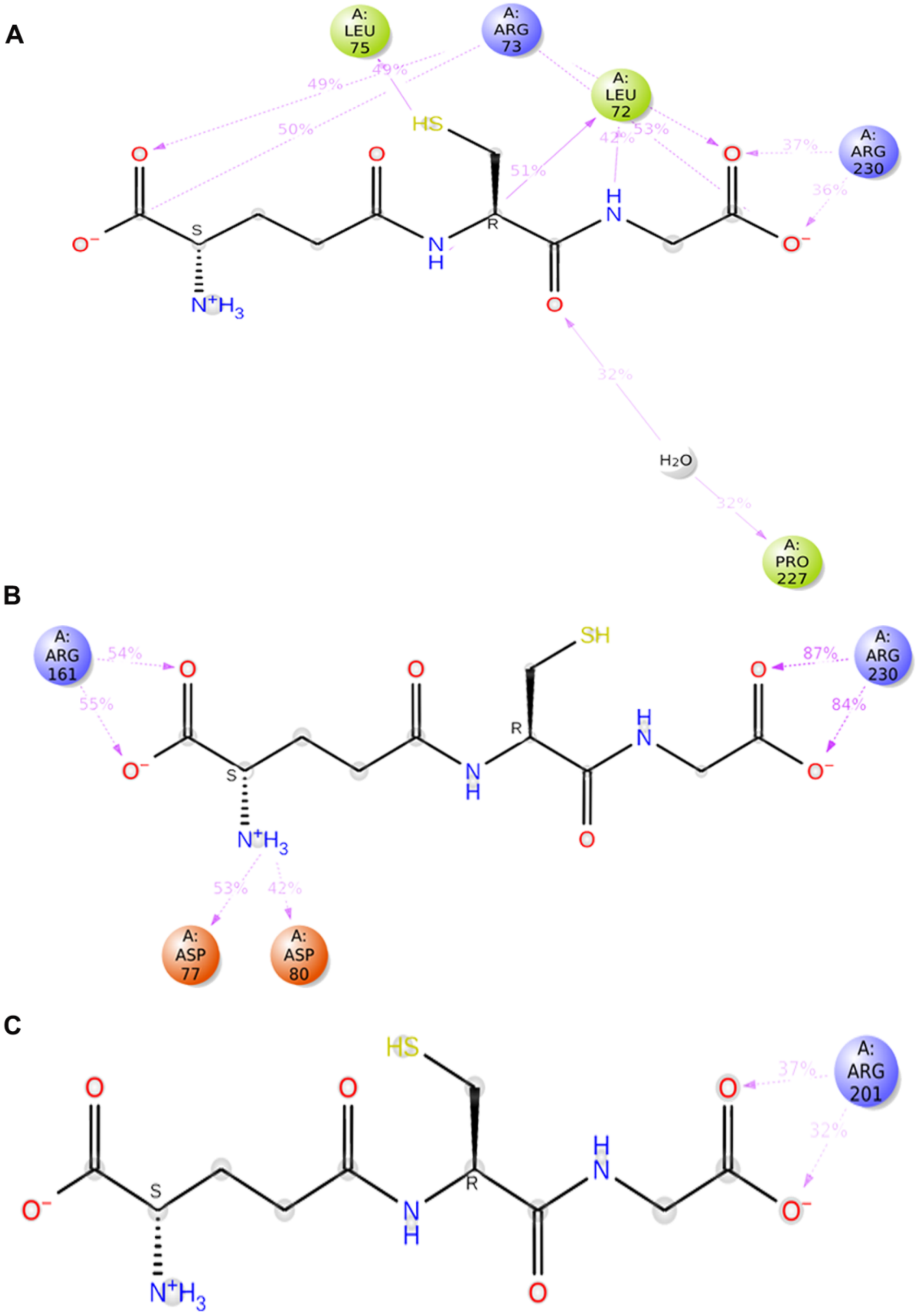

Figure S7: Interactions between GSH and the R206Q structures retained for more than 30% of the simulation time. A) R206Q run 1; B) R206Q run 2; C) R206Q run 3.

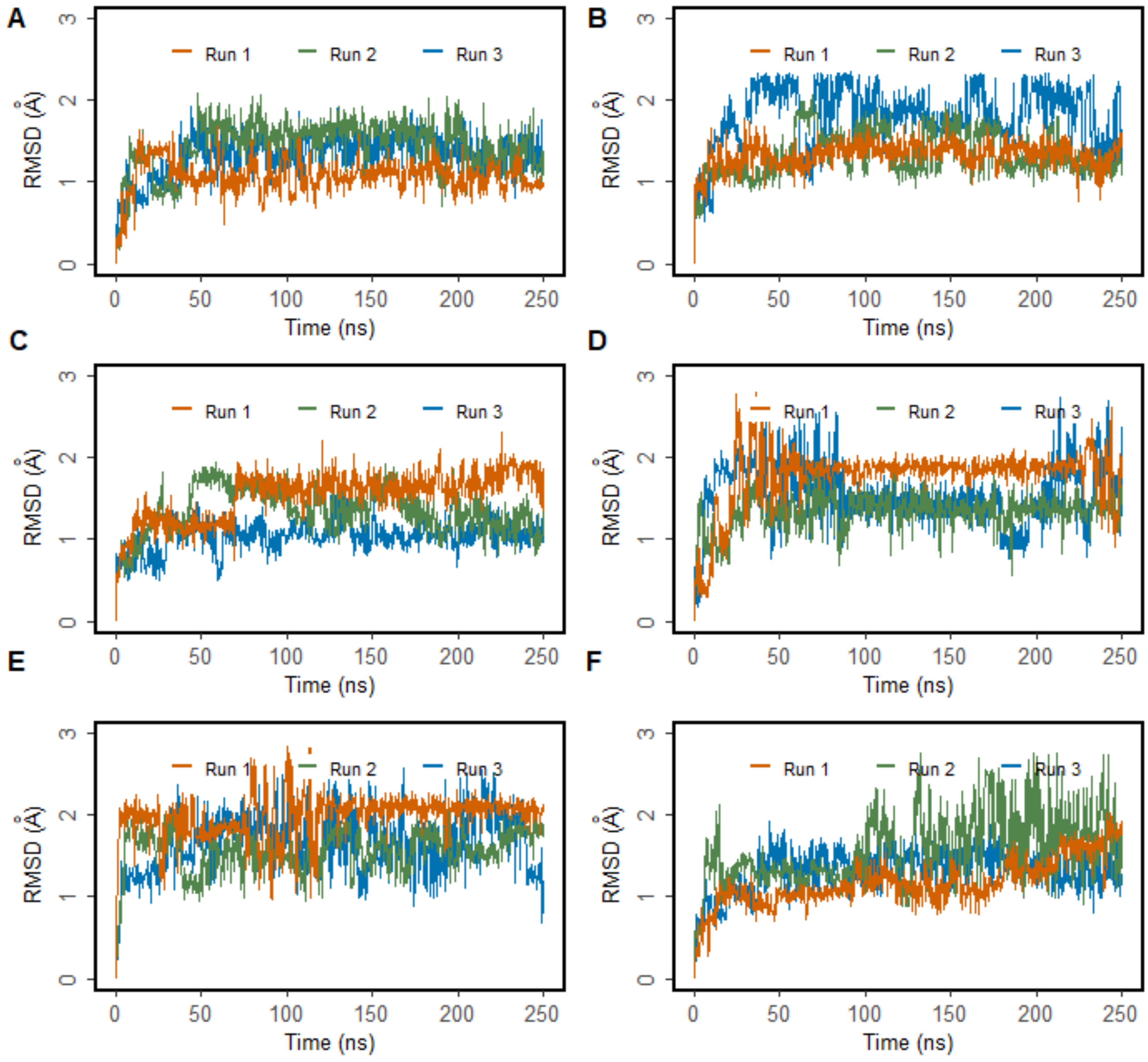

Figure S8: RMSD of GSH in 250 ns MD simulations. A) WT B) R161G C) R161Q D) R206P E) R206W and F) R206Q.

.

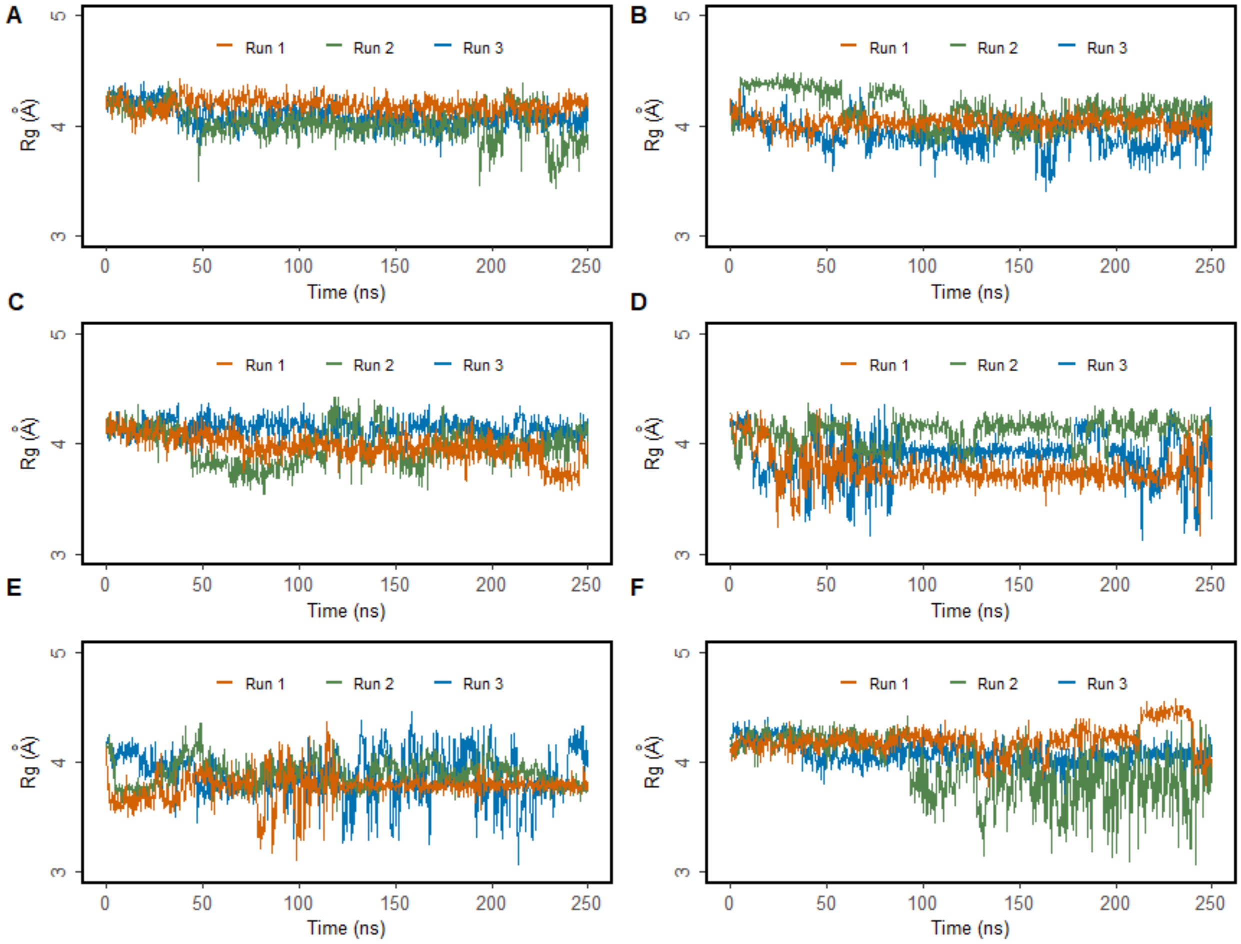

Figure S9: Radius of gyration (Rg) of GSH in 250 ns MD simulations. A) WT B) R161G C) R161Q D) R206P E) R206W and F) R206Q.

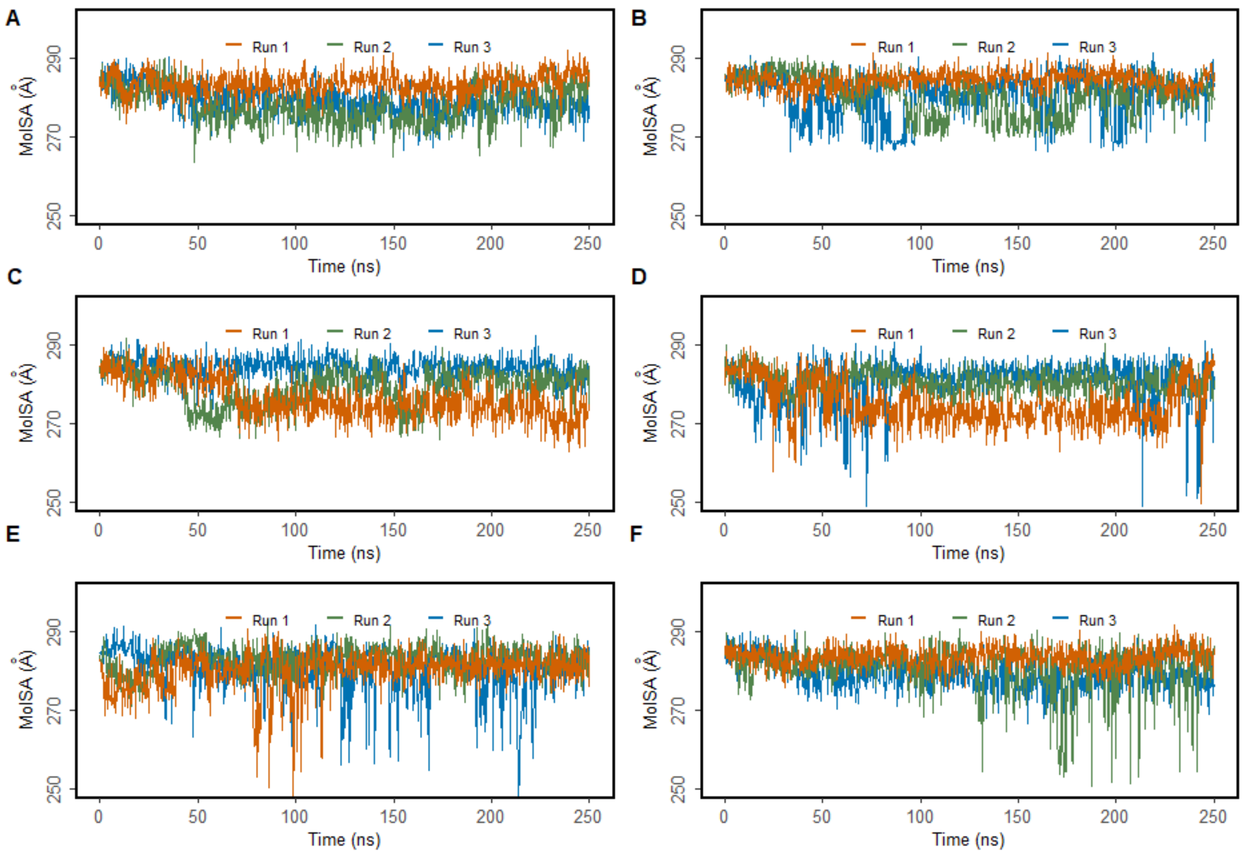

Figure S10: Molecular surface area (MolSA) of GSH in 250 ns MD simulations. A) WT B) R161G C) R161Q D) R206P E) R206W and F) R206Q.

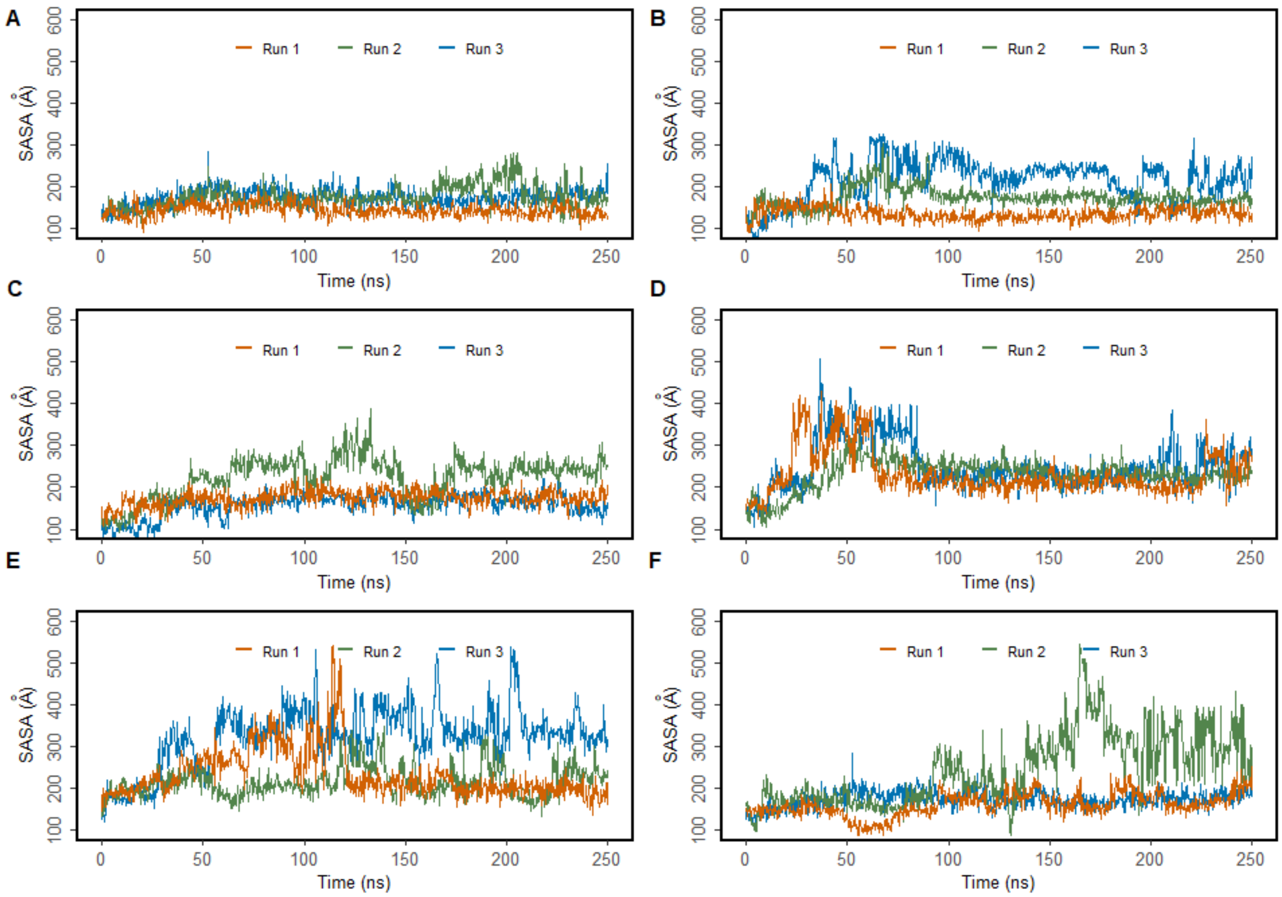

Figure S11: Solvent accessible surface area (SASA) of GSH in 250 ns MD simulations. A) WT B) R161G C) R161Q D) R206P E) R206W and F) R206Q.

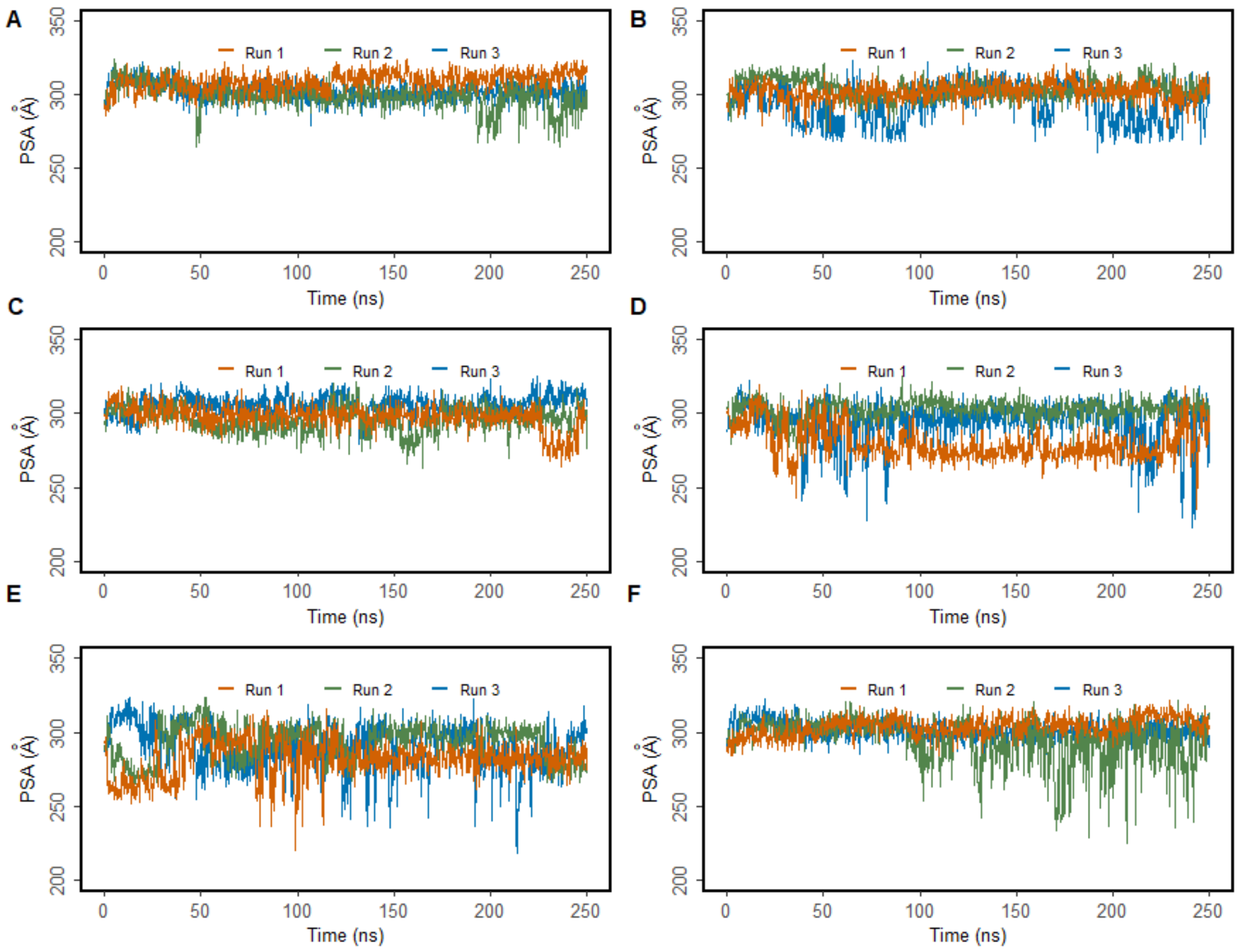

Figure S12: Polar surface area (PSA) of GSH in 250 ns MD simulations. A) WT B) R161G C) R161Q D) R206P E) R206W and F) R206Q.

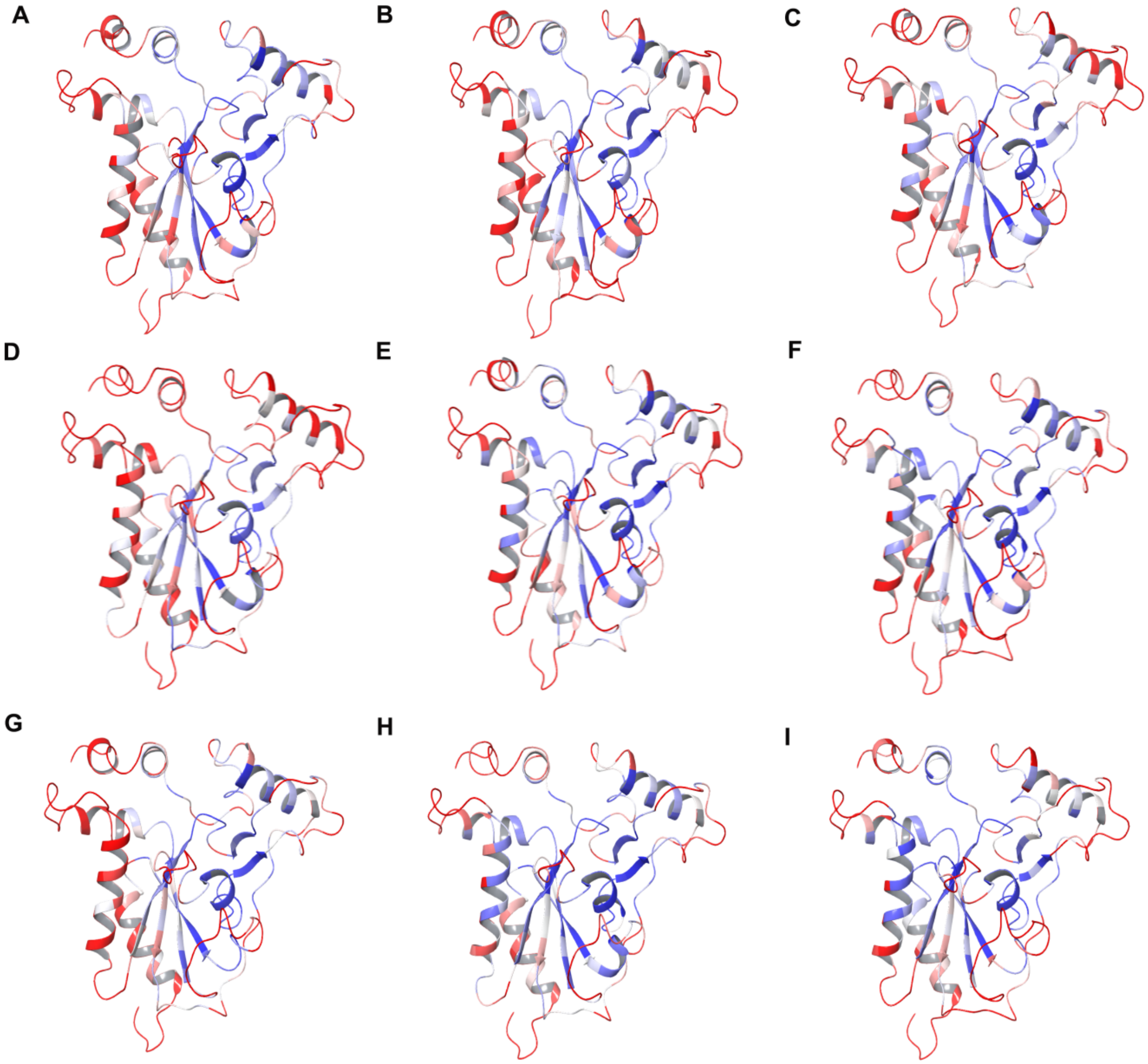

Figure S13: Protein structure rendered after RMSF from MD simulations projected as beta factors in MMACHC wild type (WT) and R161G and R161Q mutants. Red indicates regions with higher RMSF and blue denotes region with lower RMSF. A) WT run 1 B) WT run 2 C) WT run 3 D) R161G run 1 E) R161G run 2 F) R161G run 3 G) R161Q run 1 H) R161Q run 2 I) R161Q run 3.

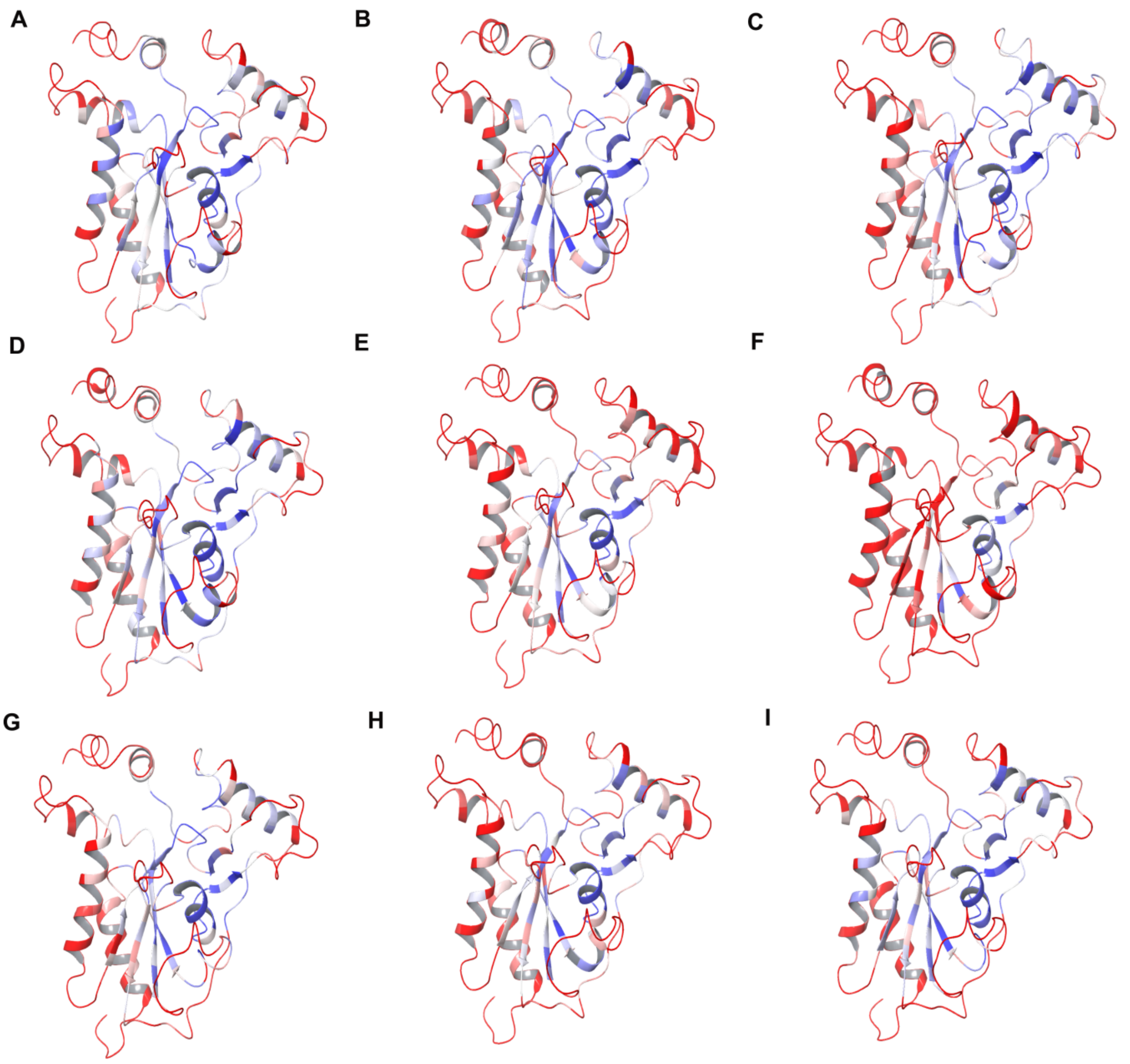

Figure S14: Protein structure rendered after RMSF from MD simulations projected as beta factors in MMACHC R206P, R206W, and R206Q mutants. Red indicates regions with higher RMSF and blue denotes region with lower RMSF. A) R206P run 1; B) R206P run 2; C) R206P run 3; D) R206W run 1; E) R206W run 2; F) R206W run 3; G) R206Q run 1; H) R206Q run 2; I) R206Q run 3.

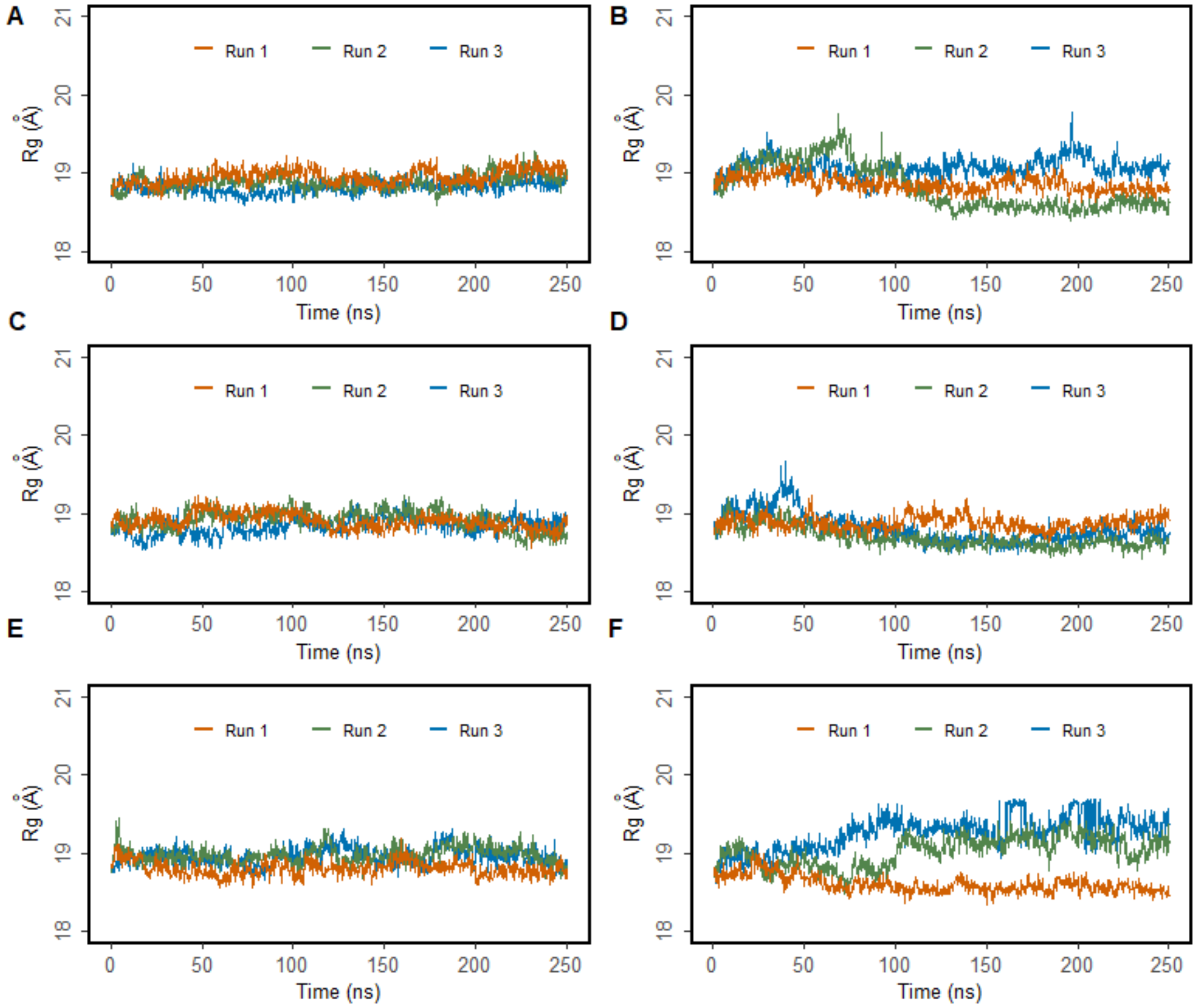

Figure S15: Radius of gyration (Rg) of MMACHC protein in 250 ns MD simulations. A) WT B) R161G C) R161Q D) R206P E) R206W and F) R206Q.

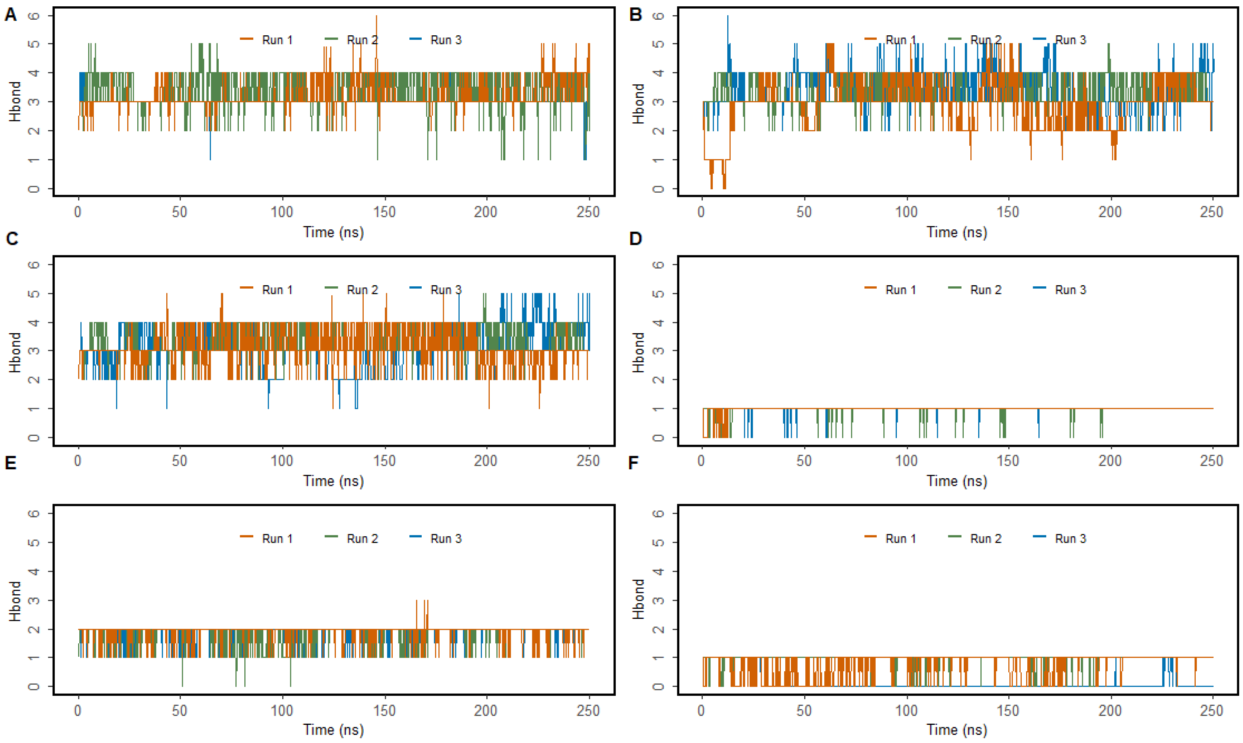

Figure S16: Number of protein-ligand hydrogen bonds observed during MD simulations in A) WT MMACHC, B) R161G mutant, C) R161Q mutant, D) R206P mutant, E) R206W mutant, F) R206Q mutant.

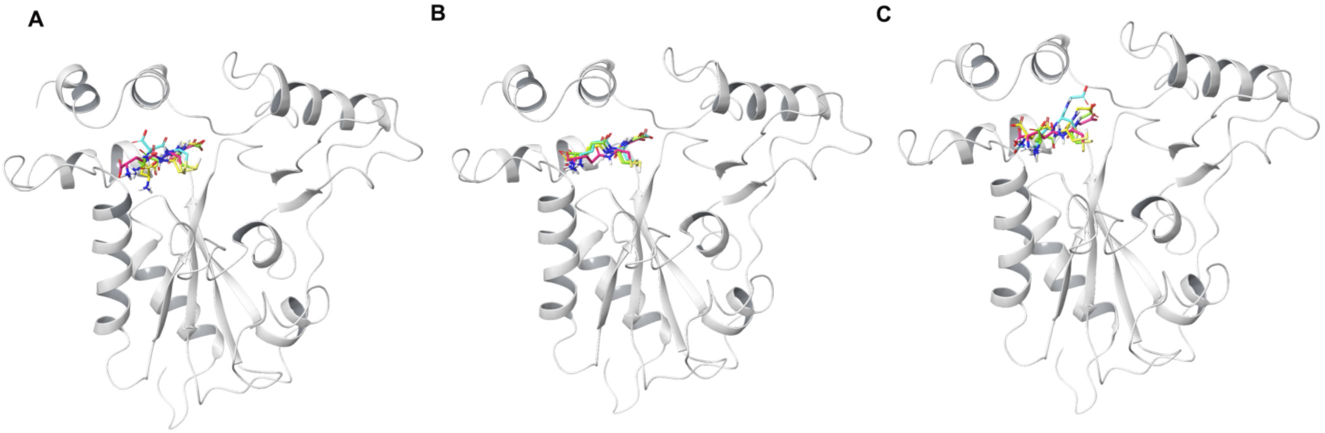

Figure S17: Snapshots of GSH taken at 0 ns (pink), 100 ns (cyan), 200 ns (yellow) and 250 ns (green) from the trajectory of GSH complexed with wild type (WT) MMACHC.

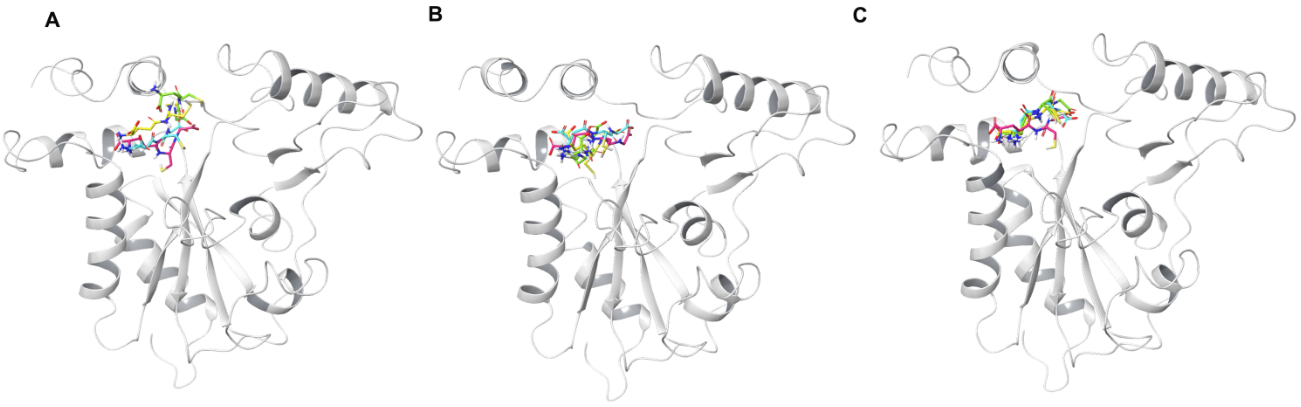

Figure S18: Snapshots of GSH taken at 0 ns (pink), 100 ns (cyan), 200 ns (yellow) and 250 ns (green) from the trajectory of GSH complexed with R161G.

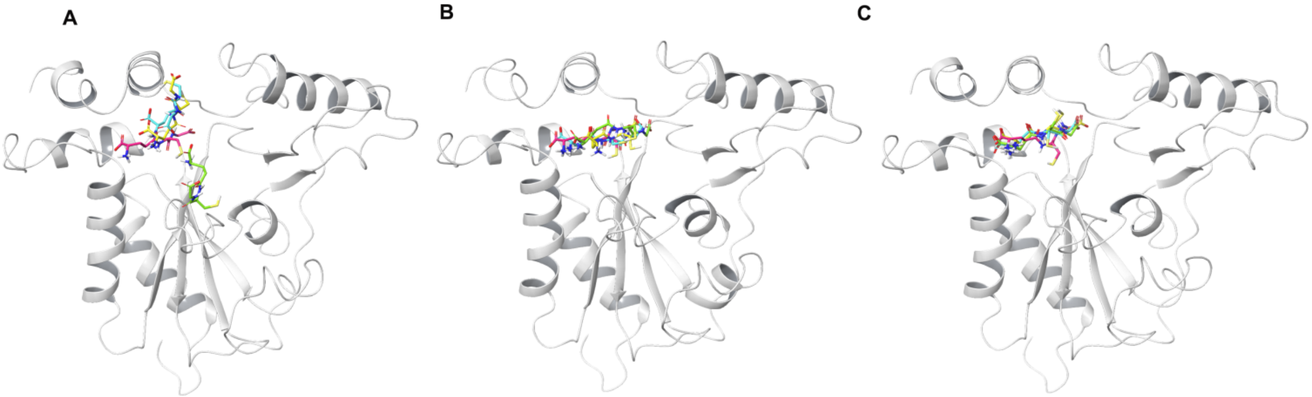

Figure S19: Snapshots of GSH taken at 0 ns (pink), 100 ns (cyan), 200 ns (yellow) and 250 ns (green) from the trajectory of GSH complexed with R161Q.

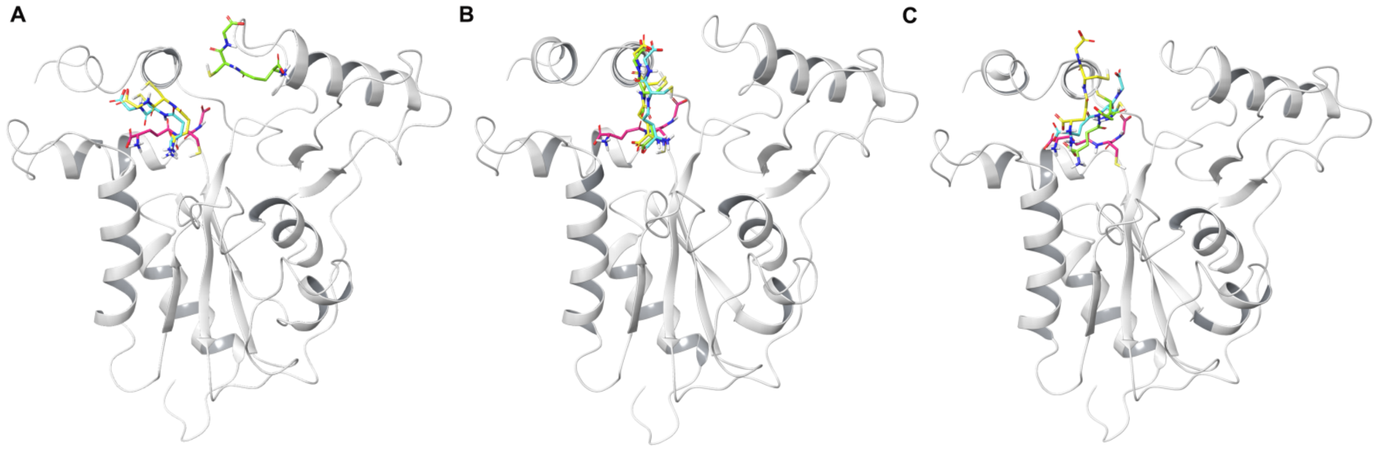

Figure S20: Snapshots of GSH taken at 0 ns (pink), 100 ns (cyan), 200 ns (yellow) and 250 ns (green) from the trajectory of GSH complexed with R206P.

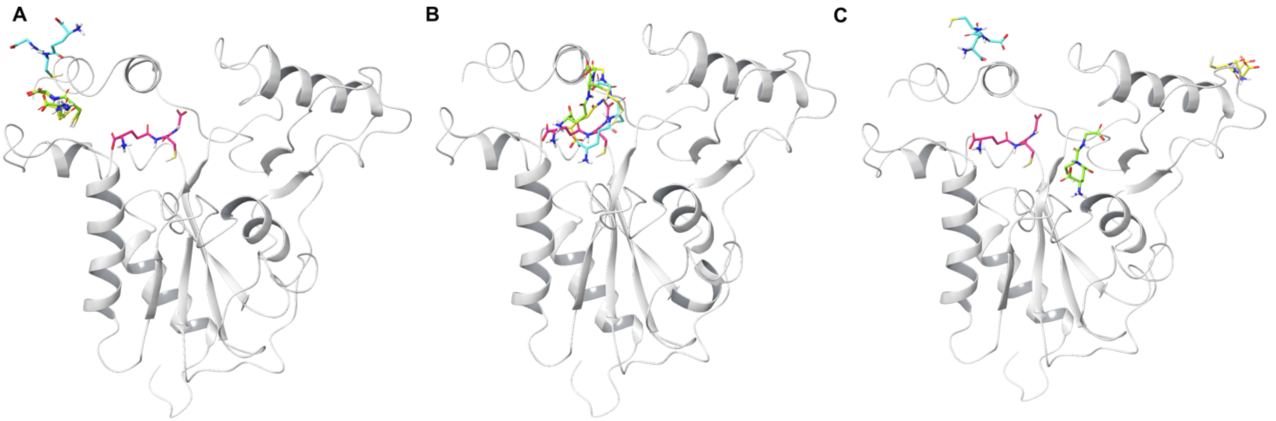

Figure S21: Snapshots of GSH taken at 0 ns (pink), 100 ns (cyan), 200 ns (yellow) and 250 ns (green) from the trajectory of GSH complexed with R206W.

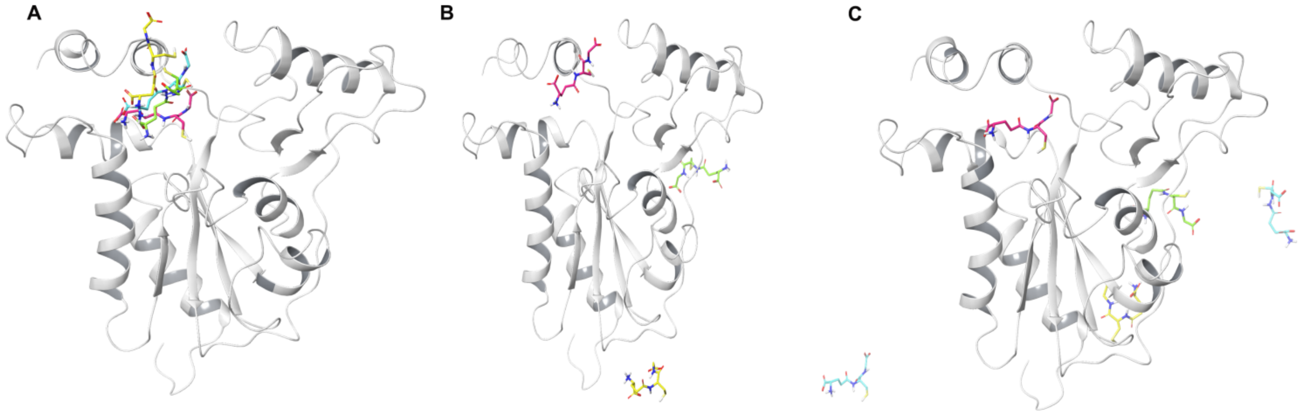

Figure S22: Snapshots of GSH taken at 0 ns (pink), 100 ns (cyan), 200 ns (yellow) and 250 ns (green) from the trajectory of GSH complexed with R206Q.

Table S1: Atom types and parameters used for MeCbl and GSH

MeCbl			
Atom	type	vdw	symbol
N1	245	N3	NI
N2	245	N3	NI
N3	245	N3	NI
N4	245	N3	NI
C5	135	C1	CT
C6	135	C1	CT
C7	135	C1	CT
C8	135	C1	CT
C9	135	C1	CT
C10	235	C2	C
O11	233	O2	O
N12	237	N1	N
C13	135	C1	CT
C14	135	C1	CT
C15	135	C1	CT
C16	235	C2	C
O17	233	O2	O
N18	237	N1	N
C19	190	C2	CD
C20	141	C3	CM
C21	135	C1	CT
C22	141	C3	CM
C23	135	C1	CT
C24	135	C1	CT
C25	135	C1	CT
C26	235	C2	C
O27	233	O2	O
N28	237	N1	N
C29	135	C1	CT
C30	135	C1	CT
C31	135	C1	CT
C32	235	C2	C
O33	233	O2	O
N34	237	N1	N
C35	190	C2	CD
C36	141	C3	CM
C37	141	C3	CM
C38	135	C1	CT
C39	135	C1	CT
C40	135	C1	CT
C41	135	C1	CT
C42	135	C1	CT
C43	135	C1	CT
C44	235	C2	C
O45	233	O2	O
N46	237	N1	N

C47	190	C2	CD
C48	141	C3	CM
C49	135	C1	CT
C50	141	C3	CM
C51	135	C1	CT
C52	135	C1	CT
C53	135	C1	CT
C54	135	C1	CT
C55	235	C2	C
O56	233	O2	O
N57	238	N1	N
C58	135	C1	CT
C59	135	C1	CT
C60	235	C2	C
O61	233	O2	O
N62	237	N1	N
C63	190	C2	CD
C64	135	C1	CT
C65	135	C1	CT
C66	135	C1	CT
O67	457	O16	OS
O68	441	O8	O2Z
O69	457	O16	OS
P70	446	P1	P1
O71	457	O16	OS
C72	135	C1	CT
C73	135	C1	CT
O74	154	O3	OH
C75	931	C1	CO
O76	180	O1	OS
C77	181	C1	CT
C78	135	C1	CT
O79	154	O3	OH
N80	2802	N1	N*
C81	2801	C4	C56A
C82	2803	C4	CRA
N83	2804	N3	N5B
C84	2805	C4	C56B
C85	2797	C4	CA
C86	2798	C4	CA
C87	135	C1	CT
C88	2799	C4	CA
C89	135	C1	CT
C90	2800	C4	CA
H91	140	H1	HC
H92	140	H1	HC
H93	140	H1	HC
H94	140	H1	HC
H95	140	H1	HC
H96	140	H1	HC

H97	140	H1	HC
H98	140	H1	HC
H99	240	H8	H
H100	240	H8	H
H101	140	H1	HC
H102	140	H1	HC
H103	140	H1	HC
H104	140	H1	HC
H105	140	H1	HC
H106	240	H8	H
H107	240	H8	H
H108	140	H1	HC
H109	140	H1	HC
H110	140	H1	HC
H111	140	H1	HC
H112	140	H1	HC
H113	140	H1	HC
H114	140	H1	HC
H115	140	H1	HC
H116	240	H8	H
H117	240	H8	H
H118	140	H1	HC
H119	140	H1	HC
H120	140	H1	HC
H121	140	H1	HC
H122	140	H1	HC
H123	240	H8	H
H124	240	H8	H
H125	146	H6	HC
H126	140	H1	HC
H127	140	H1	HC
H128	140	H1	HC
H129	140	H1	HC
H130	140	H1	HC
H131	140	H1	HC
H132	140	H1	HC
H133	140	H1	HC
H134	140	H1	HC
H135	140	H1	HC
H136	140	H1	HC
H137	240	H8	H
H138	240	H8	H
H139	140	H1	HC
H140	140	H1	HC
H141	140	H1	HC
H142	140	H1	HC
H143	140	H1	HC
H144	140	H1	HC
H145	140	H1	HC
H146	140	H1	HC

H147	140	H1	HC
H148	140	H1	HC
H149	240	H8	H
H150	140	H1	HC
H151	140	H1	HC
H152	240	H8	H
H153	240	H8	H
H154	140	H1	HC
H155	140	H1	HC
H156	140	H1	HC
H157	140	H1	HC
H158	140	H1	HC
H159	140	H1	HC
H160	140	H1	HC
H161	140	H1	HC
H162	240	H8	HO
H163	140	H1	HC
H164	140	H1	HC
H165	140	H1	HC
H166	140	H1	HC
H167	240	H8	HO
H168	146	H6	HA
H169	146	H6	HA
H170	140	H1	HC
H171	140	H1	HC
H172	140	H1	HC
H173	140	H1	HC
H174	140	H1	HC
H175	140	H1	HC
H176	146	H6	HA
Co177	868	Co	Co
C178	135	C1	CT
H179	140	H1	HC
H180	240	H8	HO
H181	140	H1	HC
H182	140	H1	HC
H183	140	H1	HC

GSH

Atom	type	vdw	symbol
N3998	286	N7	NP
C3999	951	C1	CT
C4000	952	C2	CO3
O4001	954	O9	O2Z
O4002	954	O9	O2Z
C4003	135	C1	CT
C4004	135	C1	CT
C4005	235	C2	C
O4006	233	O2	O
N4007	238	N1	N

C4008	224	C1	CT1
C4009	235	C2	C
O4010	233	O2	O
C4011	135	C1	CT
S4012	200	S2	SH
N4013	238	N1	N
C4014	135	C1	CT
C4015	271	C2	CO3
O4016	272	O2	O2Z
O4017	272	O2	O2Z
H4018	240	H8	H
H4019	240	H8	H
H4020	240	H8	H
H4021	140	H1	HC
H4022	140	H1	HC
H4023	140	H1	HC
H4024	140	H1	HC
H4025	140	H1	HC
H4026	240	H8	H
H4027	140	H1	HC
H4028	140	H1	HC
H4029	140	H1	HC
H4030	240	H8	HS
H4031	240	H8	H
H4032	140	H1	HC
H4033	140	H1	HC
